# The incidence and risk of venous thromboembolism associated with peripherally inserted central venous catheters in hospitalized patients: A systematic review and meta-analysis

**DOI:** 10.3389/fcvm.2022.917572

**Published:** 2022-07-26

**Authors:** Anju Puri, Haiyun Dai, Mohan Giri, Chengfei Wu, Huanhuan Huang, Qinghua Zhao

**Affiliations:** ^1^Department of Nursing, The First Affiliated Hospital of Chongqing Medical University, Chongqing, China; ^2^Department of Respiratory and Critical Care Medicine, The First Affiliated Hospital of Chongqing Medical University, Chongqing, China

**Keywords:** peripherally inserted central catheters, deep vein thrombosis, pulmonary embolism, meta-analysis, venous thromboembolism, central venous catheters

## Abstract

**Background:**

Venous thromboembolism (VTE) can be fatal if not treated promptly, and individual studies have reported wide variability in rates of VTE associated with peripherally inserted central catheters (PICC). We thus conducted this meta-analysis to investigate the overall incidence and risk of developing PICC-related VTE in hospitalized patients.

**Methods:**

We searched PubMed, Embase, Scopus, and Web of Science databases from inception until January 26, 2022. In studies with a non-comparison arm, the pooled incidence of PICC-related VTE was calculated. The pooled odds ratio (OR) was calculated to assess the risk of VTE in the studies that compared PICC to the central venous catheter (CVC). The Newcastle-Ottawa Scale was used to assess methodological quality.

**Results:**

A total of 75 articles (58 without a comparison arm and 17 with), including 109292 patients, were included in the meta-analysis. The overall pooled incidence of symptomatic VTE was 3.7% (95% CI: 3.1–4.4) in non-comparative studies. In the subgroup meta-analysis, the incidence of VTE was highest in patients who were in a critical care setting (10.6%; 95% CI: 5.0–17.7). Meta-analysis of comparative studies revealed that PICC was associated with a statistically significant increase in the odds of VTE events compared with CVC (OR, 2.48; 95% CI, 1.83–3.37; *P* < 0.01). However, in subgroup analysis stratified by the study design, there was no significant difference in VTE events between the PICC and CVC in randomized controlled trials (OR, 2.28; 95% CI, 0.77–6.74; *P* = 0.13).

**Conclusion:**

Best practice standards such as PICC tip verification and VTE prophylaxis can help reduce the incidence and risk of PICC-related VTE. The risk-benefit of inserting PICC should be carefully weighed, especially in critically ill patients. Cautious interpretation of our results is important owing to substantial heterogeneity among the studies included in this study.

## Introduction

The peripherally inserted central catheter (PICC) or PICC line is a 50–60 cm long, thin, flexible tube inserted in the arm vein with the tip of the catheter positioned at the lower third of the superior vena cava (1–3). PICC, which is safer to insert than traditional central venous catheters (CVCs), is frequently used in inpatient and outpatient settings (2). In recent years, PICCs have gained popularity in medical practice over other CVCs due to several advantages, including ease of insertion, safety, cost-effectiveness, multiple uses for extended periods, and a reduction in central line-associated blood stream infection (CLABSI) (3, 4). In addition, the growth of nurse-led PICC teams has made the use of PICCs more convenient and accessible in various settings (5).

Despite the aforementioned benefits, the widespread use of PICC exposes patients to a number of high-risk complications, such as the risk of VTE and CLABSI, which increase mortality, morbidity, length of hospital stay, and medical expenses (6, 7). VTE, which includes deep vein thrombosis (DVT) and pulmonary embolism (PE), is a potentially fatal condition in hospitalized patients. Accumulating evidence suggests that PICC insertion has been associated with a high risk of VTE, specifically arm DVT and PE (8–10). The documented incidence of symptomatic PICC-associated VTE ranges from 6 to 25% (9, 11, 12), while asymptomatic VTE ranges from 35 to 71.9% (13, 14).

In 2013, Chopra et al. (15) conducted a systematic review and meta-analysis to assess the risk of VTE associated with PICC. They found that the incidence of PICC-related DVT was highest in critically ill patients (13.91%), followed by cancer patients (6.67%). However, the studies in their meta-analysis varied greatly because they included many studies in which the PICC tip location was not reported. PICC tip position is an important predictor of catheter-related thrombosis, and verifying tip position at the cavoatrial junction appears to be associated with a significant decrease in DVT (16, 17). Furthermore, one-third of the studies included in the meta-analysis by Chopra et al. were only in abstract form, which may have overestimated the overall result. The results presented in conference abstracts are questionable because systematic reviewers are unable to extract sufficient information on study design, methods, risk of bias, outcomes, and detailed results (18). A recently published meta-analysis that assessed the thrombotic rate associated with PICCs in patients who had catheter insertion performed with proper tip location verification found that the pooled DVT rate was 2.4%, with the thrombotic rate being higher in onco-hematologic patients (5.9%) (19). Nonetheless, this study completely neglected retrospective studies on the same topic. As a result, these findings do not represent a reliable estimate of PICC-related thrombotic events. Despite advances in modern vascular techniques over the years, the precise estimation of the incidence and risk of VTE associated with PICC in hospitalized patients remains unclear.

Therefore, we conducted a systematic review and meta-analysis of studies published up-to-date to estimate the incidence of PICC-related VTE and compare this risk between PICC and CVCs in hospitalized patients. The objective was only to include studies in which the catheter tip position was confirmed in order to assess the precise incidence and risk of PICC-related VTE among hospital patients.

## Materials and methods

The Preferred Reporting Items for Systematic Reviews and Meta-Analyses (PRISMA) guideline was used to conduct this systemic review and meta-analysis (20). This is a systematic review and meta-analysis with only secondary data analysis, so no ethical approval or consent is required.

### Literature search and search strategies

The two authors (AP and HD) independently performed a systematic literature search of PubMed, Embase, Scopus, and Web of Science databases up to January 26, 2022, without language restriction. To include all relevant articles, the following search terms were combined with Boolean operators: PICC, peripherally inserted central venous catheter, PICC line, PICC, venous thromboembolism, pulmonary embolism, deep vein thrombosis, deep vein thrombi, and upper-extremity deep vein thrombosis. The detailed search strategy is presented in Supplementary Appendix 1. A manual reference search was also conducted to identify additional potentially eligible studies.

### Study selection and eligibility criteria

We imported all the searched articles into the EndNote X 8.0 software and excluded duplicate studies. The titles and abstracts of all articles retrieved from the search were screened independently by two authors. The same investigators reviewed the full text of potentially eligible studies. Disagreements were resolved through discussion among the authors. The inclusion criteria were as follows: (a) study population: studies with participants ≥ 18 years of age and PICC placed in veins of the upper arm, (b) The studies reporting VTE events (DVT, PE, or both) after the insertion of PICC, (c) studies in which the catheter tip position was confirmed using radiographic imaging of the chest or fluoroscopy or any other method following PICC placement. The **exclusion criteria were** as follows: (a) studies with pediatric cases or pregnant women, (b) studies reporting complications after PICC is inserted into leg veins, and studies reporting complications such as phlebitis or thrombophlebitis but not VTE. We also excluded review articles, letters, comments, case reports, editorials, and conference abstracts. All records were screened for eligible studies by two investigators (AP and HD). Disagreements between reviewers during full-text screening were resolved by consensus with a third reviewer (MG).

### Data extraction and outcome measures

Two authors independently extracted data, and a Microsoft Excel spreadsheet was used to record the data. The following data were extracted for each included study: author, publication year, geographical location, study design, sample size, number of DVT or PE or both, PICC indication, method of VTE diagnosis, and use of DVT prophylaxis. We divided the eligible studies into non-comparison studies (studies reporting PICC-related VTE occurrence in PICC recipients) and comparison studies (studies in which PICCs were compared with other CVCs in terms of venous thromboembolism). The primary outcome was the development of VTE, which included DVT and PE. DVT was defined as thrombosis of the deep veins of the upper arm detected using Doppler ultrasound, compression ultrasonography, or venography. The occurrence of PE after PICC insertion was determined based on diagnoses reported in individual studies. We contacted the study authors to obtain missing or ambiguous information.

### Quality assessment

The Newcastle–Ottawa scale was used independently by two authors (AP and MG) to assess the quality of the eligible studies. Each of the included studies was evaluated in the three domains listed below: (1) selection of exposed and non- exposed cohorts (four items: representativeness of the exposed cohort, selection of non-exposed cohort, ascertainment of the exposure, and the outcome present at the start of the study); (2) comparability (one item: comparability of cohorts based on the design of the analysis); and (3) outcome of interest (three items: assessment of outcome, length of follow-up, and adequacy of follow-up). Comparative studies with stars in all domains were deemed high quality. For non-comparison studies, the comparability of the cohort domain was excluded, and stars in all domains except comparability were deemed high quality. Studies with four to eight stars were considered moderate quality, while those with less than four stars were deemed low quality.

### Statistical analysis

Meta-analysis was conducted separately for the non-comparison and comparison studies. A Freeman-Tukey double arcsine transformation with a random effect model was used to calculate the weighted proportion of VTE with corresponding 95% confidence intervals (CIs) in non-comparison studies. The total number of PICC-associated DVTs was divided by the total number of PICCs placed to calculate the incidence of PICC-related DVTs, as some studies reported data on DVT events per PICC rather than per patient. For comparison studies, the OR with corresponding 95% CIs was calculated, and the results were pooled using the DerSimonian and Laird inverse-variance-weighted random-effects models as there was variability between included studies. The *I*^2^ statistic and Cochran’s *Q* test were used to assess heterogeneity between included studies. According to the *I*^2^ statistic, values of < 25, 25–75%, and >75% indicate low, moderate, and high levels of heterogeneity, respectively. Publication bias was assessed visually using funnel plots and quantitatively using Begg’s and Egger’s regression tests. When there was publication bias, trim and fill analysis was performed to correct the funnel plot asymmetry. Finally, a sensitivity analysis was conducted to assess the impact of each study on the overall pooled estimates by leave-one-out meta-analysis (by removing one study at a time). All tests were two-sided, and a *p*-value of less than 0.05 was considered statistically significant. All analyses were performed in the ‘‘meta’’ package using R version 4.1.0 for Windows^1^.

### Prespecified subgroup analyses

The subgroup analysis were performed according to VTE type (DVT vs. DVT/PE), setting (critical care or non-critical care), patient population (oncology or non-oncology), study design (prospective vs. retrospective), study location, DVT prophylaxis (yes or no or not reported), and publication year.

## Results

### Search results and study characteristics

**FIGURE 1:**
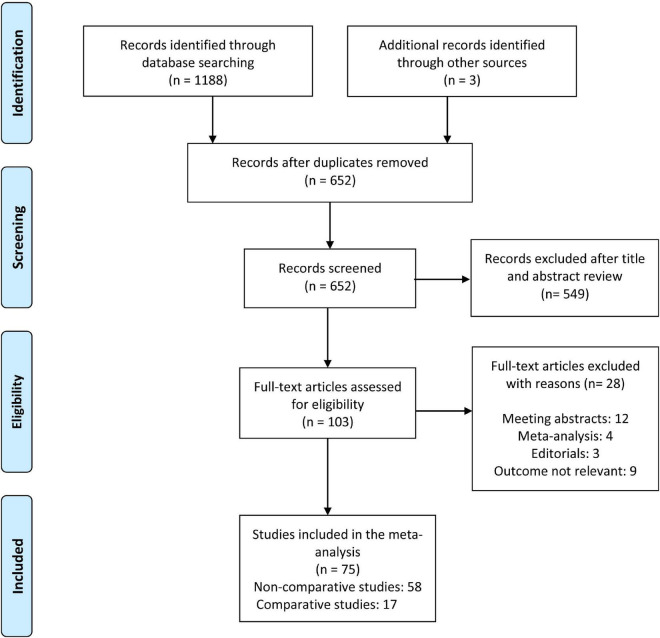
PRISMA flow chart of the study selection process.

The search yielded 1188 citations, as well as three studies identified from an additional source. After removing duplicates and screening titles and abstracts, ultimately, 103 articles were assessed for full-text review. After excluding 28 ineligible studies, a total of 75 articles were included in the meta-analysis. Figure 1 depicts the flowchart of the study selection process. Fifty eight non-comparative studies (2, 5, 7, 10, 11, 21–73) included 103351 patients who underwent 109163 PICC insertions (Table 1), while seventeen comparative studies (74–90) with 5941 patients compared PICCs with CVCs (Table 2). For non-comparison studies, only thirteen provided data on anticoagulant prophylaxis for VTE prevention (Table 1). The sample sizes ranged widely across non-comparison studies (median: 373 patients; range: 26–42,661). Among 58 non-comparative studies, 25 were conducted in the United States, 11 in China, 8 in Italy, 4 in France, and ten elsewhere. Of the 58 studies included, 35 were retrospective, and 23 were prospective studies (Table 1). The median sample size for studies with a comparison arm was 256 (range: 31–1,331). Seven of the 17 comparative studies were conducted in the United States, 3 in Italy, 2 in Canada, and 5 in other countries. Among the 17 included comparative studies, there were nine retrospective studies, five prospective RCTs, and three prospective studies (Table 2).

**TABLE 1:** Characteristics of included studies without a comparison group.

Study (Year)	Study design	Country	Total patients	Total PICC	Patient population	PICC indication	Type of VTE	VTE %	Method for VTE diagnosis	DVT prophylaxis	Study quality
Campagna et al. (65)	PC	Malaysia	26	26	Cancer	Long term antibiotic therapy (65%) and chemotherapy (35%)	DVT	38.5%	Upper limb venogram	No	High
Chasseigne et al. (64)	RC	United States	119	354	Hospitalized patients	Long-term antibiotic therapy, TPN, chemotherapy	DVT	9%	Venogram	NR	High
Aw et al. (11)	RC	Canada	340	340	Cancer	Chemotherapy	DVT	5.60%	Symptomatic testing with US	No	High
Chemaly et al. (63)	RC	Italy	57	60	Hemato-oncology	Chemotherapy, intravenous drugs, TPN	DVT	5%	Symptomatic and Doppler examination	NR	High
Chen et al. (62)	PC	Italy	291	291	Cancer	Chemotherapy, nutrition	DVT	11.7%	ultrasonography	NR	High
Chen et al. (60)	RC	Italy	1250	1250	Hospitalized patients	Intravenous drugs, TPN	DVT	2%	Symptomatic testing with compression US	NR	High
Chopra et al. (7)	PC	France	77	77	Hospitalized patients	Intravenous antibiotics, chemotherapy, TPN	DVT	1.30%	Symptomatic testing with US	NR	High
Cornillon et al. (59)	RC	United States	2063	2063	Hospitalized patients	Intravenous antibiotics	DVT, PE	1.40%	US for DVT, V/Q for PE	NR	High
DeLemos et al. (58)	RC	China	748	748	Cancer	Chemotherapy	DVT	7.40%	Symptomatic testing with US	NR	High
Evans et al. (56)	RC	China	938	938	Cancer	Chemotherapy	DVT	2.03%	Symptomatic testing with US	NR	High
Evans et al. (55)	RC	United States	23010	23010	Hospitalized patients	Various, ICU patients, Intravenous antibiotics, chemotherapy, TPN	DVT, PE	2.10%	Symptomatic testing with US	NR	High
Fletcher et al. (54)	PC	France	37	37	Patients after HSCT	Chemotherapy, hydration, Intravenous drugs	DVT	8.10%	Symptomatic testing with US	NR	High
González et al. (53)	PC	United States	33	33	Neurological ICU	Long-term venous access and CVP monitoring	DVT	3%	US	Yes	Moderate
Grove et al. (52)	PC	France	117	174	Patients with cystic fibrosis, bronchiectasis	Antibiotic courses	DVT	2.30%	Symptomatic testing with Doppler US	NR	High
Haglund et al. (50)	PC	United States	1728	2014	Hospitalized patients	Medication administration, intravenous antibiotic, venous access	DVT	3.00%	Symptomatic testing with US	Yes	High
Jianning et al. (49)	PC	United States	1758	1827	Hospitalized patients	Medication administration, intravenous antibiotic, venous access, Chemotherapy	DVT	1.90%	Symptomatic testing with venous duplex ultrasound	Yes	High
Jones et al. (48)	RC	United States	479	479	Neurological ICU	Medication administration, Venous access	DVT, PE	8.10%	Symptomatic testing with US	Yes	High
Kang et al. (47)	PC	Spain	1142	1142	Hospitalized patients	Chemotherapy, TPN, antibiotics	DVT	2.01%	Symptomatic testing with US	No	High
King et al. (46)	RC	United States	678	813	Hospitalized patients	Intravenous access, antibiotics, chemotherapy, TPN	DVT	3.90%	Symptomatic testing with US	NR	High
Lambrechts et al. (44)	RC	United States	129	129	Hospitalized patients	Intravenous drugs	DVT, PE	3.10%	Symptomatic testing with US, V/Q for PE	NR	High
Li et al. (43)	PC	China	406	406	Cancer	Chemotherapy	DVT	3.20%	Symptomatic testing with US	No	High
Liang et al. (42)	RC	United Kingdom	490	490	Cancer	Chemotherapy, TPN, antibiotics	DVT	5.51%	Symptomatic testing with US	No	High
Liu et al. (41)	PC	China	477	477	Cancer	Chemotherapy, TPN, antibiotics	DVT	1.90%	Symptomatic testing with US	NR	Moderate
Lobo et al. (40)	RC	United States	896	1296	Hospitalized patients	Intravenous access, antibiotics, chemotherapy, TPN	DVT	2.10%	Symptomatic testing with US	Yes	Moderate
Maneval et al. (39)	PC	United States	42687	42687	Hospitalized patients	Intravenous access, antibiotics	DVT	1.40%	Symptomatic testing with US	Yes	High
Mariggiò et al. (38)	RC	United States	660	660	Orthopedics surgery	Intravenous access, antibiotics	DVT	3.18%	Symptomatic testing with compression US	yes	High
Mermis et al. (37)	RC	China	2353	2353	Cancer	Chemotherapy	DVT	7.01%	Symptomatic testing with US	NR	High
Merrell et al. (36)	RC	China	1363	1363	Cancer	Chemotherapy	DVT	5.60%	Symptomatic testing with compression US	NR	High
Liem et al. (10)	PC	United States	690	2056	Hospitalized patients	Various intravenous access, antibiotics	DVT	2.60%	Symptomatic testing with US	Yes	High
Meyer et al. (35)	PC	China	104	104	Cancer	Chemotherapy	DVT	1.92%	Symptomatic testing with US	Yes	High
Nash et al. (34)	RC	United States	777	954	Hospitalized patients	Intravenous access, antibiotics, chemotherapy, TPN	DVT, PE	4.89%	Symptomatic testing with US	No	High
Ong et al. (33)	PC	United States	203	203	Acute care or critical setting	Intravenous access, antibiotics, chemotherapy	DVT	6.40%	US	NR	High
Pittiruti et al. (32)	PC	Italy	100	100	Hospita;ized HSCT patients	Various intravenous access	DVT	9%	Symptomatic testing with US	NR	High
McDiarmid et al. (5)	RC	Canada	656	656	Hospitalized patients	Antibiotics, chemotherapy, TPN	DVT	1.52%	Symptomatic testing with US	NR	Moderate
Pittiruti et al. (31)	RC	United States	117	369	Hospitalized cystic fibrosis patients	Intravenous antibiotics	DVT	7.60%	Symptomatic testing with compression US	yes	High
Poletti et al. (30)	PC	United States	460	389	Hospitalized patients	Intravenous access, antibiotics, chemotherapy, TPN	DVT	0.50%	Symptomatic testing with US	NR	High
Rabinstein et al. (29)	RC	United States	1307	879	Hospitalized patients	Intravenous access	DVT	3.40%	Symptomatic testing with US	NR	High
Mielke et al. (2)	RC	Germany	484	522	Cancer	Intravenous access, antibiotics, chemotherapy, TPN	DVT	2.90%	Symptomatic testing with US	NR	High
Sato et al. (28)	RC	Canada	147	376	Cystic fibrosis patients	Intravenous access	DVT	4.50%	12 symptomatic patients duplex US, 5 asymptomatic patients contrast venography	NR	High
Seeley et al. (27)	RC	Australia	317	2882	Hospitalized patients	Chemotherapy, TPN	DVT	2.60%	Symptomatic testing with US	NR	Moderate
Sharp et al. (26)	RC	Italy	65	65	Critically ill/ICU	Intravenous access, TPN, CVP monitoring	DVT	3.10%	Symptomatic testing with US	Yes	High
Sperry et al. (25)	PC	Italy	180	180	cancer	Chemotherapy	DVT	0.50%	Symptomatic testing with US	NR	High
Tian et al. (24)	RC	Italy	137	137	Acute Cardiac care or critical setting	Venous access	DVT	1.40%	Symptomatic testing with US	NR	High
Tran et al. (23)	RC	United States	62	62	Neuro ICU	Intravenous access, TPN, CVP monitoring	DVT	29%	Symptomatic testing with compression US	NR	Moderate
Trerotola et al. (22)	RC	Japan	85	118	Cancer	Fluid replacement, Intravenous nutrition, antibiotics	DVT	1.70%	Symptomatic testing with US	NR	High
Vidal et al. (21)	RC	United States	233	233	Hospitalized patients	Intravenous access, various reasons	DVT	7.30%	Symptomatic testing with US	NR	Moderate
Walshe et al. (73)	PC	Australia	136	136	Cancer	Chemotherapy, antibiotics	DVT, PE	2.90%	Symptomatic testing with US	Yes	High
Wang et al. (72)	RC	United States	672	798	Hospitalized patients	Intravenous access, antibiotics, TPN	DVT	1.30%	Symptomatic testing with US	No	High
Wilson et al. (71)	RC	China	161	165	Cancer	Chemotherapy	DVT	0.61%	Symptomatic testing with US	NR	Moderate
Xing et al. (70)	RC	United States	498	899	Cancer	Chemotherapy in patients with hematological malignancies	DVT	4.30%	Symptomatic testing with US	No	High
Zerla et al. (61)	PC	United States	50	50	Critically ill/ICU	Intravenous access, antibiotics, TPN, chemotherapy	DVT	52%	Symptomatic patients venography, asymptomatic patients compression US	NR	High
Zhang et al. (51)	PC	France	115	127	Hospitalized patients	Intravenous antibiotics, TPN, chemotherapy	DVT	2.40%	Symptomatic testing with US	NR	Moderate
Abdullah et al. (69)	PC	United States	320	351	Cancer	Intravenous antibiotics, TPN, intravenous access, chemotherapy	DVT	3.40%	Symptomatic testing with US	NR	High
Allen et al. (68)	RC	China	2247	2315	Cancer	INTRAVENOUS antibiotics, TPN, chemotherapy	DVT	5.70%	Symptomatic testing with US	NR	High
Bellesi et al. (67)	RC	United States	431	431	Critically ill/ICU	Various, ICU patients	DVT	8.40%	Symptomatic testing with US	Yes	High

**TABLE 2:** Characteristics of included studies with a comparison group.

Study (Year)	Study design	Country	Total patients	Patient population	Method for VTE diagnosis	DVT prophylaxis	VTE events in both groups				Study quality

							**PICC group**		**Comparison group**		
							**VTE events**	**Total PICC**	**VTE events**	**Total PICC**	
Bertoglio et al. (66)	RC	China	187	188	Cancer	Chemotherapy	DVT	2.10%	Symptomatic testing with US	NR	High
Dupont et al. (57)	PC	Italy	30	30	Cancer	Chemotherapy	DVT	0	US	NR	High
Kleidon et al. (45)	RC	China	8028	8028	Cancer	Chemotherapy	DVT	3.10%	Symptomatic testing with US	NR	High
Akhtar and Lee (74)	RC	Canada	305	Cancer	Symptomatic testing with US	NR	37	408	5	72	High
Bonizzoli et al. (75)	PC	Italy	239	Critical care or ICU	Symptomatic testing with US	Yes	31	114	12	125	High
Picardi et al. (90)	PC (RCT)	United States	152	Neuro-ICU	Symptomatic testing with US	NR	4	72	0	80	High
Refaei et al. (76)	PC (RCT)	France	256	Cancer	Symptomatic testing with US	NR	7	128	5	128	High
Ryu et al. (77)	RC	Italy	178	Cancer	Symptomatic testing with US	NR	6	130	1	48	High
Sriskandarajah et al. (78)	RC	United States	31	Burn ICU	Symptomatic testing with US	Yes	1	36	0	82	High
Taxbro et al. (79)	PC (RCT)	United States	80	Neuro-ICU	Symptomatic testing with US	NR	17	39	9	41	High
Wilson et al. (80)	PC	United States	184	Surgical ICU	Symptomatic testing with US	Yes	18	89	41	265	High
Worth et al. (81)	RC	United States	371	ICU	Symptomatic testing with US	NR	8	200	2	200	High
							**PICC group**		**Comparison group**		
							**VTE events**	**Total PICC**	**VTE events**	**Total PICC**	
Smith et al. (82)	PC (RCT)	Italy	93	AML or cancer	Symptomatic testing with US	NR	1	46	5	47	High
Brandmeir et al. (83)	RC	Canada	1331	Hematology oncology	Symptomatic testing with US	NR	82	338	41	325	High
Clatot et al. (84)	RC	Korea	430	Acute care/ICU	Symptomatic testing with US	NR	1	97	0	333	High
Nolan et al. (89)	RC	United States	838	Hospitalized patients	Symptomatic testing with US	NR	14	555	2	283	High
Corti et al. (85)	RC	United Kingdom	583	hemato-oncology	Symptomatic testing with US	NR	20	346	4	237	High
Fearonce et al. (86)	PC (RCT)	Sweden	399	Cancer	Symptomatic testing with US	NR	16	201	2	198	High
Fletcher et al. (87)	RC	United States	572	Neuro-ICU	Symptomatic testing with US	NR	36	431	2	141	High
Malinoski et al. (88)	PC	Australia	66	Hemato oncology	Symptomatic testing with US	NR	14	75	2	31	Moderate

PICC, peripherally inserted central catheter; VTE, venous thromboembolism; PC, prospective cohort study; RC, retrospective cohort study; RCT, randomized controlled trial; NR, not reported; US, ultrasound; ICU, intensive care unit; AML, acute myeloid leukemia.

### Quality of the studies

The quality of the included comparison and non-comparison studies was evaluated using the Newcastle-Ottawa scale. Sixteen of the 17 comparison studies were of high quality, while one was of moderate quality. Of the 58 non-comparison studies, 49 were of high quality, and nine were of moderate quality (Supplementary Table 1).

### Meta-analysis of the incidence of peripherally inserted central catheters-related venous thromboembolism

**FIGURE 2:**
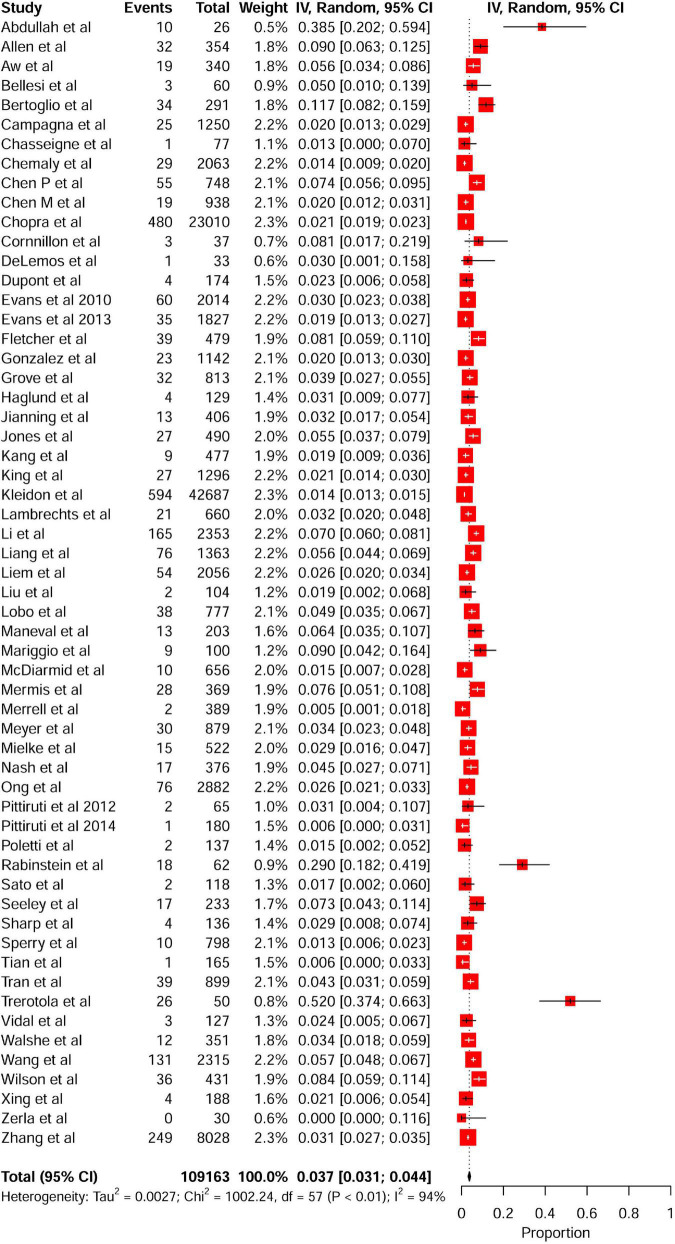
Forest plot showing the pooled incidence of PICC-related VTE among hospitalized patients in non-comparative studies. PICC, peripherally inserted central catheter; VTE, venous thromboembolism.

**FIGURE 3:**
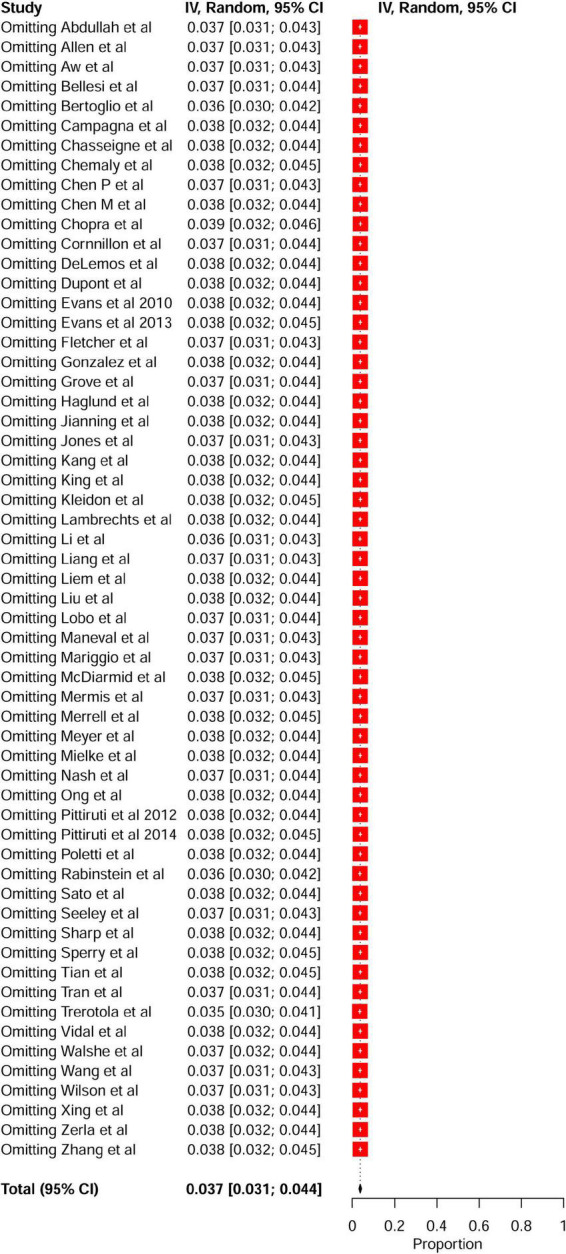
Leave-one-out sensitivity analysis of the pooled incidence of PICC-related VTE among hospitalized patients. PICC, peripherally inserted central catheter; VTE, venous thromboembolism.

Fifty-eight studies reported VTE incidence among patients after PICC insertion, and the overall pooled incidence of VTE was 3.7% (95% CI: 3.1–4.4), with high heterogeneity (*I*^2^ = 94%, *P* < 0.01) (Figure 2). Visual inspection of asymmetry in the funnel plot (Supplementary Figure 1) and the Egger’s test (*t* = 5.29; *P* < 0.001) revealed evidence of publication bias. The trim-and-fill method was used to account for publication bias in these studies, and the funnel plot became symmetrical after imputing 24 unpublished studies (Supplementary Figure 2). Furthermore, sensitivity analysis revealed the robustness of our finding, with the recalculated pooled incidence of VTE after excluding each study ranging from 3.5% (95% CI: 3.0–4.1) to 3.9% (95% CI: 3.2–4.6) (Figure 3).

A subgroup meta-analysis was also performed to analyze the incidence of PICC-related VTE based on the study setting, patient population, type of VTE, DVT prophylaxis, study design, study location, and publication year. Subgroup analyses based on the study setting showed that the incidence of VTE in critical care or intensive care unit (ICU) settings was 10.6% (95% CI: 5.0–17.7), whereas the incidence of VTE in non-critical care or non-ICU patients was 3.3% (95% CI: 2.7–3.8) (Table 3 and Supplementary Figure 3). On subgroup analysis stratified by patient population, the pooled incidence of VTE was 3.9% (95% CI: 2.9–4.9) in oncology patients and 3.5% for non-oncology patients (95% CI: 2.9–4.2) (Table 3 and Supplementary Figure 4). When data were analyzed based on the type of VTE, the pooled incidence of DVT was 3.9% (95% CI: 3.1–4.7), while the incidence of DVT/PE was 3.4% (95% CI: 2.0–5.2) (Table 3 and Supplementary Figure 5). The subgroup analysis of DVT prophylaxis revealed similar pooled proportions of VTE among three different subgroups, i.e., the pooled incidence of VTE was 3.4% (95% CI: 2.3–4.7) in studies that reported DVT prophylaxis, 3.8% (95% CI: 3.0–4.7) in studies that did not report DVT prophylaxis, and 4.2% (95% CI: 2.5–6.3) in studies that used no DVT prophylaxis (Table 3 and Supplementary Figure 6). Subgroup analyses stratified by study design showed that the incidence of PICC-related VTE was 4% (95% CI: 3.3–4.7) in retrospective studies and 3.5% (95% CI: 2.4–4.7) in prospective studies (Table 3 and Supplementary Figure 7). According to subgroup analyses stratified by study location, the incidence of PICC-related VTE was 3.7% (95% CI: 3.1–4.3) in non-Asian studies and 3.8% (95% CI: 2.6–5.2) in Asian studies (Table 3 and Supplementary Figure 8). Further subgroup analysis stratified by publication year indicated that the incidence of VTE was 4.4% (95% CI: 3.0–6.0) in studies published from 1990 to 2010 and 3.6% (95% CI: 2.9–4.3) in studies published from 2011 to 2022 (Table 3 and Supplementary Figure 9). The summary results of the overall and subgroup meta-analyses are presented in Table 3.

**TABLE 3:** Overall and subgroup meta-analysis for incidence of VTE associated with PICC.

Variable	No. of studies	Total participants	VTE incidence (95% CI)	*I*^2^, %	Subgroup difference
**Overall analysis of VTE**	58	109163	3.7 (3.1–4.4)	94	−
Subgroup analysis for VTE					
**Setting**					
Critical care or ICU	8	1460	10.6 (5.0–17.7)	92	*P* < 0.01
Non-critical care or non-ICU	50	107703	3.3 (2.7–3.8)	94	
**Patient population**					
Oncology	23	20528	3.9 (2.9–4.9)	89	*P* = 0.54
Non-oncology	35	88635	3.5 (2.9–4.2)	93	
**Type of VTE**					
Deep vein thrombosis	52	82569	3.9 (3.1–4.7)	95	*P* = 0.5
Deep vein thrombosis and PE	6	26594	3.4 (2.0–5.2)	92	
**DVT prophylaxis**					
Yes	13	52157	3.4 (2.3–4.7)	94	*P* = 0.72
No	8	4878	4.2 (2.5–6.3)	89	
Not reported	27	52128	3.8 (3.0–4.7)	93	
**Study type**					
Retrospectives studies	35	56246	4.0 (3.3–4.7)	93	*P* = 0.77
Prospective studies	23	52917	3.5 (2.4–4.7)	92	
**Study location**					
Asian studies	13	17229	3.8 (2.6–5.2)	92	*P* = 0.89
Non-Asian studies	45	91934	3.7 (3.1–4.3)	93	
**Publication year**					
Published from 1990 to 2010	16	12815	4.4 (3.0–6.0)	93	*P* = 0.35
Published from 2011 to 2022	42	96448	3.6 (2.9–4.3)	95	

I^2^, index to quantify the degree of heterogeneity; PE, pulmonary embolism; VTE, venous thromboembolism; ICU, intensive care unit.

### Meta-analysis of the risk of venous thromboembolism associated with peripherally inserted central catheters

**FIGURE 4:**
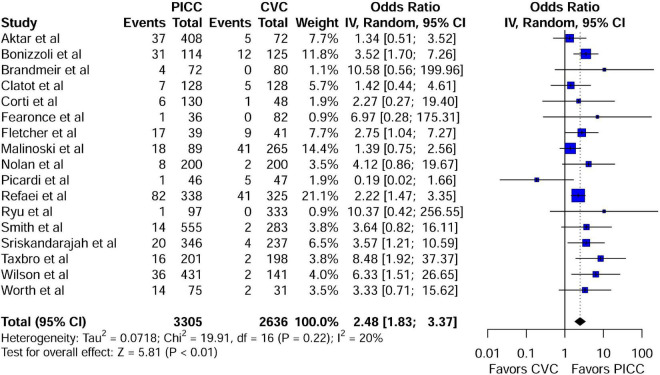
Forest plot depicting the risk of VTE between the peripherally inserted central catheter and central venous catheters in studies with a comparison arm. PICC, peripherally inserted central catheter; CVC, central venous catheter.

**FIGURE 5:**
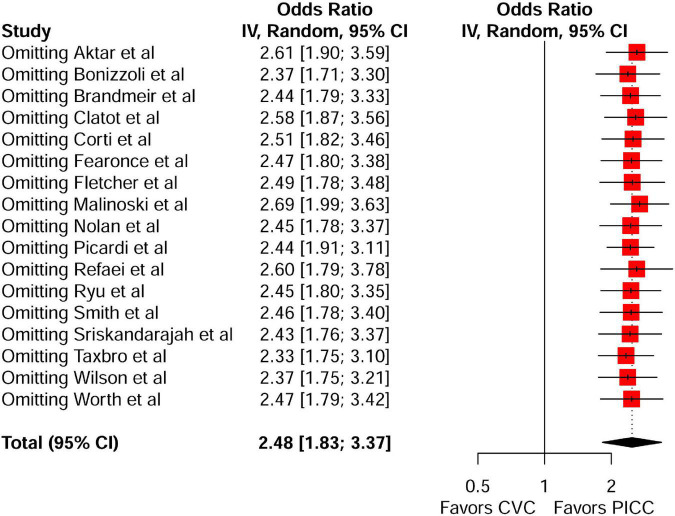
Leave-one-out sensitivity analysis of the risk of VTE between the peripherally inserted central catheter and central venous catheters in studies with a comparison arm. VTE, venous thromboembolism; PICC, peripherally inserted central catheter; CVC, central venous catheter.

A meta-analysis of 17 comparison studies showed that PICC was associated with a statistically significant increase in the odds of VTE events when compared with CVC (OR, 2.48; 95% CI, 1.83–3.37; *P* < 0.01) (Figure 4). No significant heterogeneity was detected across the included studies (*I*^2^ = 20%, *P* = 0.22) (Figure 4). Visual inspection of funnel plot symmetry (Supplementary Figure 10) followed by the Egger test (*t* = 1.3; *P* = 0.21) revealed no publication bias. The leave-one-out sensitivity analyses showed that the pooled estimated OR for VTE associated with PICC ranged between 2.33 (95% CI: 1.75–3.1) and 2.69 (95% CI: 1.99–3.63), indicating that individual studies had no significant effect on estimates (Figure 5).

Subgroup analyses were performed according to study setting, patient population, DVT prophylaxis, study design, study location, and publication year. According to subgroup analysis stratified by study setting, PICC was associated with a statistically significant increase in the odds of VTE events in both critical care/ICU (OR, 2.84; 95% CI: 1.78–4.52; *P* < 0.01) and non-critical care/non-ICU (OR, 2.24; 95% CI: 1.43–3.52; *P* < 0.01) subgroups (Table 4 and Supplementary Figure 11). Similarly, on subgroup analysis stratified by patient population, PICC was associated with a significant increase in the odds of VTE events in oncology (OR, 2.15; 95% CI: 1.31–3.53; *P* < 0.01) and non-oncology (OR, 2.76; 95% CI: 1.84–4.14; *P* < 0.01) subgroups (Table 4 and Supplementary Figure 12). Furthermore, a subgroup analysis by DVT prophylaxis revealed a significant difference in VTE events between the PICC and CVC in studies where DVT prophylaxis was not reported (OR, 2.59; 95% CI: 1.82–2.59; *P* < 0.01). However, in studies where VTE prophylaxis was reported, there was no significant difference in VTE events between the PICC and CVC (OR, 2.31; 95% CI: 1.02–5.19; *P* = 0.04) (Table 4 and Supplementary Figure 13). Subgroup analysis stratified by study design revealed that there were significant differences in the VTE events between PICC and CVC in retrospective (OR, 2.49; 95% CI: 1.80–3.33; *P* < 0.01) and prospective studies (OR, 2.30; 95% CI: 1.14–4.65; *P* = 0.02) but not in RTCs (OR, 2.28; 95% CI: 0.77–6.74; *P* = 0.13) (Table 4 and Supplementary Figure 14). Subgroup analysis by study location revealed that the American studies had a significantly higher risk of PICC-related VTE than the non-American studies (OR, 2.62; 95% CI: 1.56–4.41 vs. OR, 2.41; 95% CI: 1.59–3.67; *P* < 0.01) (Table 4 and Supplementary Figure 15). Furthermore, subgroup analysis by publication year (studies published from 2011 to 2022 and studies published from 1990 to 2010) revealed significant differences in the risk of VTE between PICC and CVC in both subgroups (*P* < 0.01) (Table 4 and Supplementary Figure 16).

**TABLE 4:** Summary of meta-analysis results by subgroups for comparative studies.

Variable	No. of studies	PICC total patients	CVC total patients	OR (95% CI)	*I*^2^, %	Meta-analysis *P*-value	Subgroup difference
**Setting**							
Critical care or ICU	8	3305	2636	2.84 (1.78–4.52)	20	<0.01	*P* = 0.48
Non-critical care or non-ICU	9	2227	1369	2.24 (1.43–3.52)	28	<0.01	
**Patient population**							
Oncology	8	1672	1086	2.15 (1.31–3.53)	34	<0.01	*P* = 0.44
Non-oncology	9	3305	1550	2.76 (1.84–4.14)	8	<0.01	
**DVT prophylaxis**							
Yes	3	239	472	2.31 (1.02–5.19)	53	0.04	*P* = 0.80
Not reported	14	3066	2164	2.59 (1.82–3.69)	15	<0.01	
**Study type**							
Retrospectives studies	9	2541	1721	2.49 (1.80–3.44)	0	<0.01	*P* = 0.97
Prospective studies	3	278	421	2.30 (1.14–4.65)	51	0.02	
Randomized controlled trials	5	486	494	2.28 (0.77–6.74)	59	0.13	
**Study location**							
Outside America	10	1883	1544	2.41 (1.59–3.67)	30	<0.01	*P* = 0.81
Conducted in America	7	1422	1092	2.62 (1.56–4.41)	15	<0.01	
**Publication year**							
Between 2011 to 2022	14	2639	2240	2.43 (1.71–3.45)	31	<0.01	*P* = 0.43
Between 1990 to 2010	3	666	396	3.73 (1.35–10.33)	0	<0.01	

I^2^, index to quantify the degree of heterogeneity; PICC, peripherally inserted central catheter; CVC, central venous catheter; ICU, intensive care unit.

## Discussion

To the best of our knowledge, this systematic review and meta-analysis assessing the incidence and risk of VTE associated with PICC in the hospitalized patient is the largest to date, with more than three times the number of patients included in any previous meta-analysis (15, 19). Furthermore, we only included studies in which the catheter tip location was confirmed in order to determine the exact incidence and risk of PICC-related VTE in hospitalized patients. This meta-analysis demonstrated that in non-comparison studies, the overall incidence of PICC-related symptomatic VTE was 3.7% (95% CI: 3.1–4.4) in hospitalized patients and 10.6% in critically ill or ICU patients. Nevertheless, there is significant heterogeneity across studies, most likely due to differences in study design, high variability in sample size, study location, measurement instruments, and timing of outcome measurements. The meta-analysis by Chopra et al. (15) showed that the weighted incidence of PICC-related symptomatic VTE was 4.30%. Moreover, the incidence of PICC-associated VTE was higher in critically ill patients (13.91%). In this study, we found a lower incidence of PICC-related VTE than in the meta-analysis by Chopra et al. (15). The possible reasons for this finding are as follows: (i) Catheter-related complications are highly dependent on the insertion technique, and the majority of the studies included by Chopra et al. (15) differed substantially in terms of insertion technique. In this meta-analysis, studies only with proper PICC tip location verification were included, so the incidence of VTE was lower than in the meta-analysis by Chopra et al. (15). (ii) Furthermore, their meta-analysis included studies that differed significantly in terms of approach to VTE diagnosis (symptomatic vs. asymptomatic DVT), catheter insertion techniques and outcomes that could have affected pooled estimates (15). (iii) The study by Chopra et al. (91) was conducted in 2012, PICC-related complications have been reduced in recent years due to the use of evidence-based intervention techniques and vascular access specialist team approach. We included 42 studies published after 2011 (i.e., the modern vascular access era) in the pooled analysis, which may have resulted in a lower incidence of PICC-related VTE. A recent meta-analysis by Balsorano et al. (19) investigated the thrombotic rate associated with PICCs and found that the pooled DVT rate was 2.4%, with the thrombotic rate being higher in onco-hematologic patients (5.9%). Although this meta-analysis included patients for whom catheter insertion was performed with ultrasound guidance and proper tip location verification, there were some limitations, including a search strategy period limited to studies published between January 2010 and November 2018. Furthermore, they excluded retrospective studies and limited their research to only prospective studies.

A meta-analysis of comparative studies in this study showed that PICCs were associated with an increased risk of VTE compared with CVCs. A similar finding was also observed for PICC-related VTE risk in a study by Chopra et al. (15). This finding is consistent with prior studies, which showed that PICCs were associated with an increased risk of VTE compared with CVCs (78–80). A previous meta-analysis found that the pooled OR for PICC-related VTE was 2.55 (95% CI: 1.54–4.23). The present study also showed a comparable odds ratio (OR, 2.48; 95% CI: 1.83–3.37). Ample evidence suggests that risk factors such as a recent cancer diagnosis, PICC size, a prior history of PICC-related venous thrombosis or DVT, obesity, catheter tip location, and chemotherapeutic agents infused through the PICC line are linked to PICC-related VTE (9, 43, 62). Although the exact mechanisms underlying catheter-related thrombosis are unclear, increased cross-sectional diameter of the peripheral veins occupied by PICC may result in venous stasis (92). Additionally, catheter misplacement or migration of the catheter tip may also cause vascular endothelium damage (93).

In light of the increased risk of DVT, it is unclear whether PICC recipients should receive pharmacological DVT prophylaxis on a regular basis. A meta-analysis of 15 RCTs of anticoagulant prophylaxis in patients with CVCs revealed that anticoagulant prophylaxis effectively prevents catheter-associated DVT (94). However, its efficacy in preventing symptomatic VTE and PE remains unknown. Similarly, a recent meta-analysis concluded that pharmacological DVT prophylaxis reduces the risk of both asymptomatic and clinically detected VTE (95). Notably, in this study, there was no significant difference in VTE risk between the PICC and CVC in the subgroup analysis (OR, 2.31; 95% CI: 1.02–5.19; *P* = 0.04). This finding is consistent with a previous meta-analysis, which found no statistically significant difference in the risk of VTE associated with PICC (15). Similarly, the evidence-based guidelines for the management of VTE published by the American Society of Hematology recommend against routine VTE prophylaxis in patients in long-term care or outpatients with low VTE risk (96). However, pharmacological VTE prophylaxis is recommended in acutely or critically ill inpatients with acceptable bleeding risk.

Of note, subgroup analysis based on the study design showed that there was no significant difference in VTE events between PICC and CVC in prospective studies (OR, 2.30; 95% CI: 1.14–4.65; *P* = 0.02) and RCTs (OR, 2.28; 95% CI: 0.77–6.74; *P* = 0.13) included in this meta-analysis. Retrospective studies are prone to selection and recall biases, which can impact on overall results. Our findings support the need for future well-designed prospective studies and RCTs to elucidate the true picture of the risk of VTE associated with PICC.

### Limitations and strengths

This meta-analysis has several limitations. First, due to the lack of a comparison group in the majority of included studies, we were unable to estimate pooled ORs of PICC-related VTE in those studies. Second, the inclusion of retrospective studies in this meta-analysis could have resulted in selection and recall biases. However, more prospective studies were included in this meta-analysis than in previous meta-analyses. Third, despite using subgroup analysis, there was substantial heterogeneity in studies with a non-comparison arm. Heterogeneity was due to differences in the study design, population, and other basic characteristics of the studies. Additionally, we could not conduct a meta-regression analysis to account for heterogeneity due to insufficient data. Fourth, many primary studies did not report data on VTE prophylaxis. Therefore, our finding regarding the use of VTE prophylaxis cannot be interpreted as a generalizable estimate.

Despite the aforementioned limitations, this meta-analysis has several strengths. First, this systematic review and meta-analysis is the most comprehensive to date because it includes the largest number (*n* = 75) of studies (both comparison and non-comparison studies) investigating the incidence and risk of VTE associated with PICCs in hospitalized patients. Second, in this meta-analysis, we included five comparative prospective RCTs, whereas previous meta-analyses did not include any RCTs. Third, this study only included studies published in peer-reviewed journals, whereas previous meta-analyses included one-third of the studies only in abstract form. Finally, sensitivity analysis of both comparison and non-comparison studies showed that our findings are robust.

### Current clinical challenges and future research

Peripherally inserted central catheters -related VTE rates were extremely variable in individual studies included in this meta-analysis due to wide variation in the study population, study design, VTE diagnostic method, and definition of VTE events across studies (28, 29, 31, 45). The majority of primary studies ignored catheter size, and the use of ultrasound for PICC insertion was inconsistent in many studies. Because the majority of PICC-related thrombotic events occur within 2–3 weeks of PICC insertion (97), future RCTs should include preplanned follow-up for catheter-related complications to reduce the impact of attrition bias in true risk detection for thrombotic events. Future studies with adequate follow-up duration, uniform definitions of VTE, and standardized risk assessment for PICC-related VTE are required. Low-molecular-weight heparins (LMWHs) are used to prevent both primary and secondary catheter-related thrombosis. The clinical practice guidelines for the management of catheter-related thrombosis from the International Initiative on Thrombosis and Cancer (ITAC), the American Society of Clinical Oncology (ASCO), the American Society of Hematology (ASH), and the National Comprehensive Cancer Network (NCCN) all recommend anticoagulation for at least 3 months or as long as the catheter is kept in place (98). However, in patients who are at high risk of bleeding, catheter removal should be considered instead of using anticoagulation for the treatment of catheter-related thrombosis (98). There are several active ongoing clinical trials (ClinicalTrials.gov) on thromboprophylaxis for VTE (for e.g., NCT04924322, NCT05029063). A recently published two-center prospective TRIM-line pilot trial (99) enrolled 105 patients with active cancer and a newly inserted CVC demonstrated that symptomatic thrombotic complications occurred in 5.8% of the rivaroxaban group compared to 9.4% of the control group. The major symptomatic VTE rate in the rivaroxaban and control groups was 3.9 and 9.4%, respectively. The findings of this study revealed that thrombotic complications are common in patients with cancer and a newly inserted CVC. The ongoing phase III study (NCT05029063) is recruiting 1828 participants to determine the efficacy and safety of rivaroxaban for preventing VTE in cancer patients following the insertion of CVC. The primary outcome measure for this study is the number of major VTE in the patient population and major bleeding episodes. Secondary outcomes include fatal VTE, a composite of major VTE and major bleeding, and proximal upper and lower extremity CVC-related DVT. The results of this trial are expected in late 2026. This study is expected to provide important data on the prevention of VTE. Additionally, future research is required to improve strategies for addressing current clinical challenges in this patient population, such as diagnostic and therapeutic management and risk stratification.

## Conclusion

In conclusion, this meta-analysis showed that the overall incidence of PICC-related symptomatic VTE was 3.7% in hospitalized patients. This incidence rate was higher in critically ill or ICU patients. PICCs are associated with an increased risk of VTE when compared to the CVC. Clinicians should perform a comprehensive risk-benefit analysis before PICC placement in critically ill patients. Further research is needed to address the heterogeneity and limitations of current evidence and to better understand the risk of VTE associated with PICC.

## Data availability statement

The original contributions presented in this study are included in the article/Supplementary material, further inquiries can be directed to the corresponding author/s.

## Author contributions

AP, HD, and QZ: conceptualization. AP, HD, MG, and CW: data curation. AP, HD, HH, and MG: formal analysis and visualization. AP, HD, and CW: investigation. AP, HD, and QZ: project administration. QZ and AP: supervision. QZ, MG, AP, and HD: validation and writing. AP, HD, CW, HH, QZ, and MG: review and editing. All authors contributed to the article and approved the submitted version.

## Footnotes

1
https://www.r-project.org/
