# Supplementary material for: The incidence and risk of venous thromboembolism associated with peripherally inserted central venous catheters in hospitalized patients: A systematic review and meta-analysis
Source: Front Cardiovasc Med. 2022 Jul 26;9:917572. doi: 10.3389/fcvm.2022.917572 (PMC9360324; doi:10.3389/fcvm.2022.917572)
Supplement: Supplementary file 1 [file Data_Sheet_1.docx]

**Supplementary Material**

**Contents**

**Supplementary Appendix 1.** The complete search strategies employed for all databases

**Supplementary Figure 1.** Funnel plots to detect publication bias among non-comparative studies

**Supplementary Figure 2.** The trim-and-fill method to account for publication bias among non-comparative studies

**Supplementary Figure 3**. Forest plot showing the incidence of VTE associated with PICC, stratified by study setting (Non-critical care/non-ICU vs. Critical care/ICU)

**Supplementary Figure 4.** Forest plot depicting the incidence of PICC-related VTE stratified by patient population (Oncology vs. non-oncology patients)

**Supplementary Figure 5.** Forest plot showing the incidence of VTE associated with PICC based on the type of VTE (DVT vs. DVT/pulmonary embolism)

**Supplementary Figure 6**. Forest plot for the incidence of VTE associated with PICC stratified by DVT prophylaxis ((DVT prophylaxis not used vs. DVT prophylaxis not reported vs. DVT prophylaxis used)

**Supplementary Figure 7.** Forest plot depicting the incidence of PICC-related VTE stratified by study design Retrospective vs. prospective studies)

**Supplementary Figure 8**. Forest plot depicting the incidence of PICC-related VTE according to study location (Asian vs. non-Asian studies)

**Supplementary Figure 9.** Forest plot showing the incidence of VTE associated with PICC, stratified by publication year (studies published from 2011 to 2022 vs. studies published from 1990 to 2010)

**Supplementary Figure 10.** Funnel plot to detect publication bias for studies comparing VTE risk between PICC and CVC

**Supplementary Figure 11.** Forest plot showing the risk of VTE associated with PICC based on the study setting (Non-critical care/non-ICU vs. Critical care/ICU)

**Supplementary Figure 12.** Forest plot showing the risk of VTE associated with PICC stratified by patient population (Oncology vs. non-oncology patients)

**Supplementary Figure 13.** Forest plot showing the risk of VTE associated with PICC stratified by DVT prophylaxis (DVT prophylaxis not reported vs. DVT prophylaxis reported)

**Supplementary Figure 14.** Forest plot depicting the risk of PICC-related VTE stratified by study design (Retrospective vs. prospective vs. randomized controlled trials)

**Supplementary Figure 15.** Forest plot depicting the risk of PICC-related VTE associated stratified by study location (studies conducted outside America vs. studies conducted in America)

**Supplementary Figure 16.** Forest plot depicting the risk of PICC-related VTE associated stratified by publication year (studies published from 2011 to 2022 vs. studies published from 1990 to 2010)

**Supplementary Table 1.** Quality assessments of included studies using the Newcastle-Ottawa scale.

**Supplementary Appendix 1- The complete search strategies employed for all databases**

**1. PubMed**

| ***Search ID*** | ***Search Terms*** |
| --- | --- |
| #1 | Peripherally inserted central catheter[Title/Abstract] OR peripherally inserted central catheter*[Title/Abstract] OR peripherally inserted central venous catheter*[Title/Abstract] OR PICC line*[Title/Abstract] OR PICC[Title/Abstract] |
| #2 | Venous thromboembolism[Title/Abstract] OR pulmonary embolism[Title/Abstract] OR deep vein thrombosis [Title/Abstract] OR deep vein thromb*[Title/Abstract] OR Upper-extremity deep vein thrombosis[Title/Abstract] |
| #3 | #1 AND #2 ( Results retrieved n= 181) |

**2. Embase**

| ***Search History*** | ***Searches*** |
| --- | --- |
| 1 | 'peripherally inserted central catheter':ab,ti OR 'peripherally inserted central catheter*':ab,ti OR 'peripherally inserted central venous catheter*':ab,ti OR 'picc line*':ab,ti OR picc:ab,ti |
| 2 | 'venous thromboembolism':ab,ti OR 'pulmonary embolism':ab,ti OR 'deep vein thrombosis':ab,ti OR 'deep vein thromb*':ab,ti OR 'upper-extremity deep vein thrombosis':ab,ti |
| 3 | #1 AND #2 ( Results retrieved n= 406) |

**3. Scopus**

( TITLE-ABS-KEY ( ( "peripherally inserted central catheter" OR "peripherally inserted central catheter*" OR "peripherally inserted central venous catheter*" OR "PICC line*" OR "PICC" ) ) AND TITLE-ABS-KEY ( ( "venous thromboembolism" OR "pulmonary embolism" OR "deep vein thrombosis" OR "deep vein thromb*" OR "Upper-extremity deep vein thrombosis" ) ) ) ( Results retrieved n= 396)

**4. Web of Science**

| ***Search History*** | ***Searches*** |
| --- | --- |
| #1 | “peripherally inserted central catheter” OR “peripherally inserted central catheter*” OR “peripherally inserted central venous catheter*” OR “PICC line*” OR “PICC” (Topic) |
| #2 | “venous thromboembolism” OR “pulmonary embolism” OR “deep vein thrombosis” OR “deep vein thromb*” OR “Upper-extremity deep vein thrombosis” (Topic) |
| #3 | #1 AND #2 ( Results retrieved n= 205) |

**Supplementary Figure 1. Funnel plot to detect publication bias among non-comparative studies.** Asymmetry in the funnel plot indicated publication bias among the included studies.


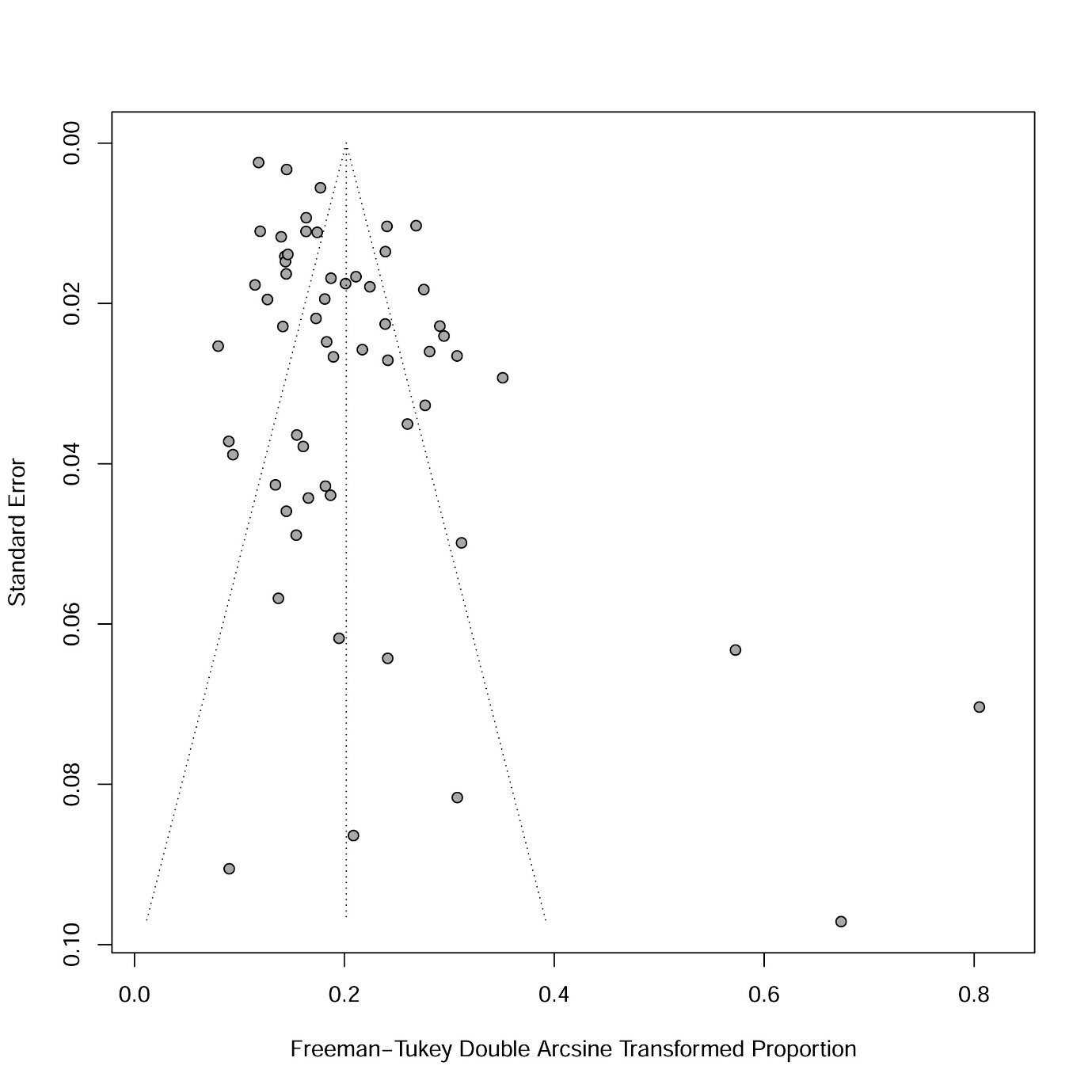


**Supplementary Figure 2. The trim-and-fill method to account for publication bias among non-comparative studies**. When the trim-and-fill method was used, twenty-four studies (white circles) were imputed, resolving the issue of publication bias among included studies (symmetrical funnel plot was obtained).

**
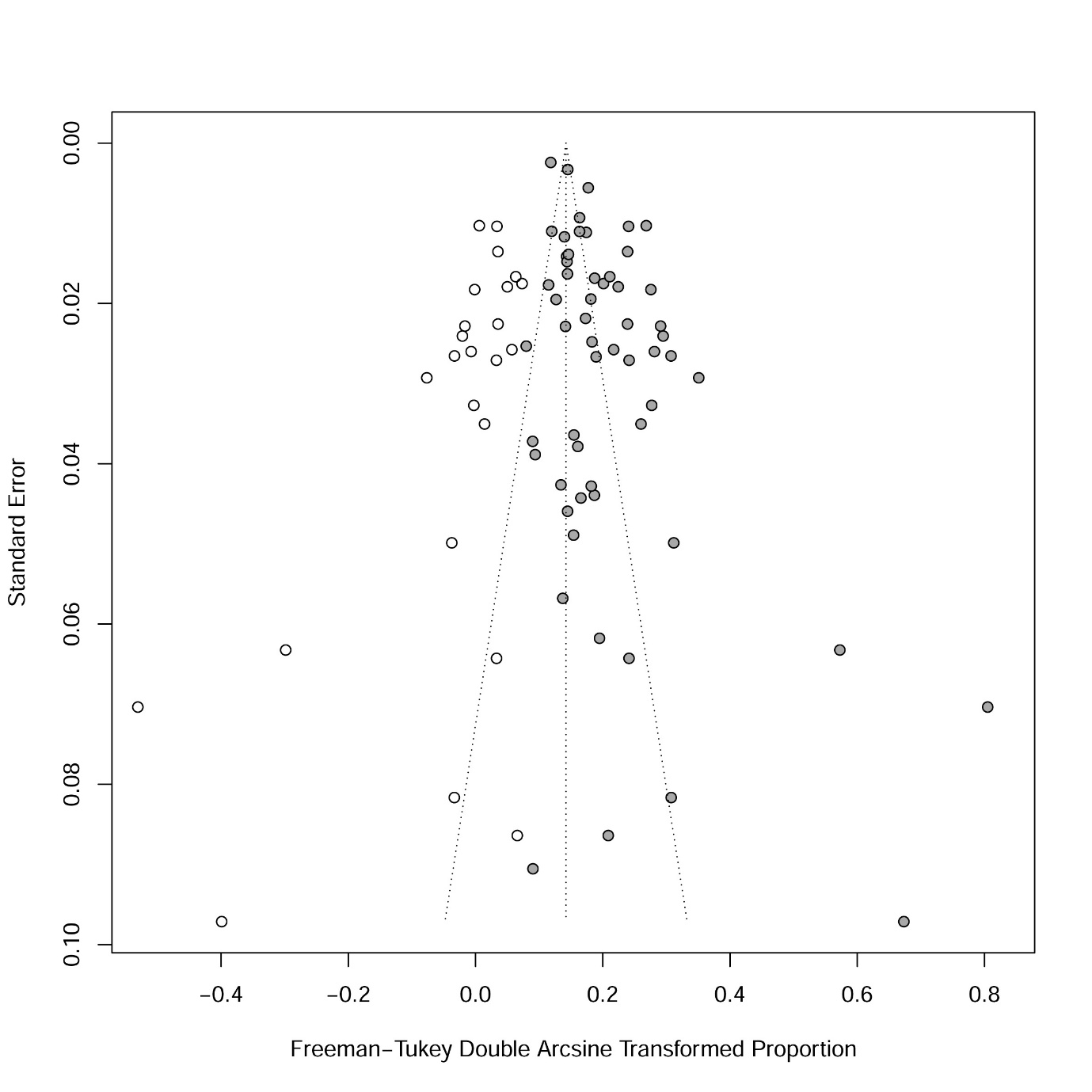
**

**Supplementary Figure 3**. **Forest plot showing the incidence of VTE associated with PICC, stratified by study setting**

**
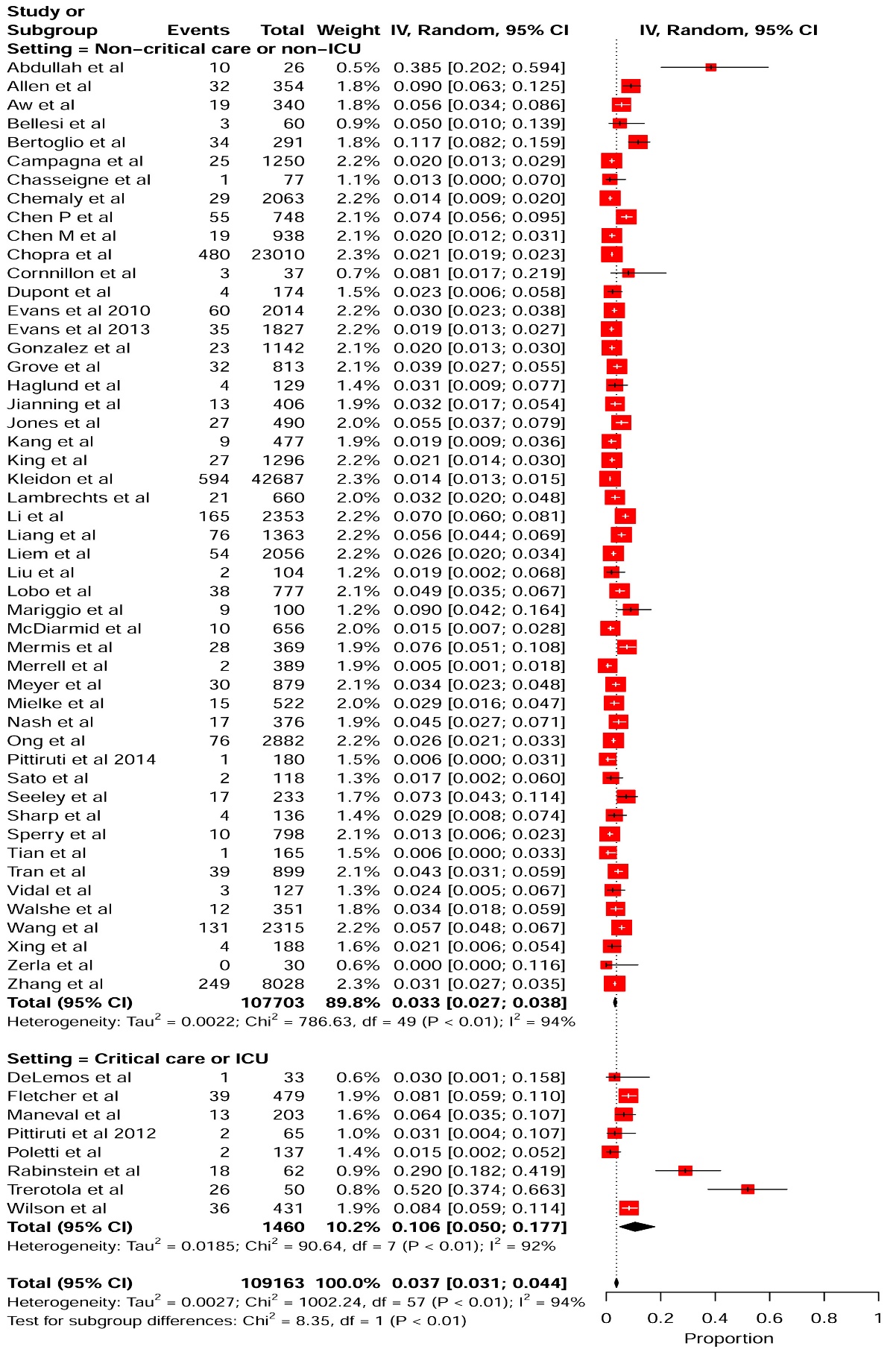
**

**Supplementary Figure 4. Forest plot depicting the incidence of PICC-related VTE stratified by patient population**

**
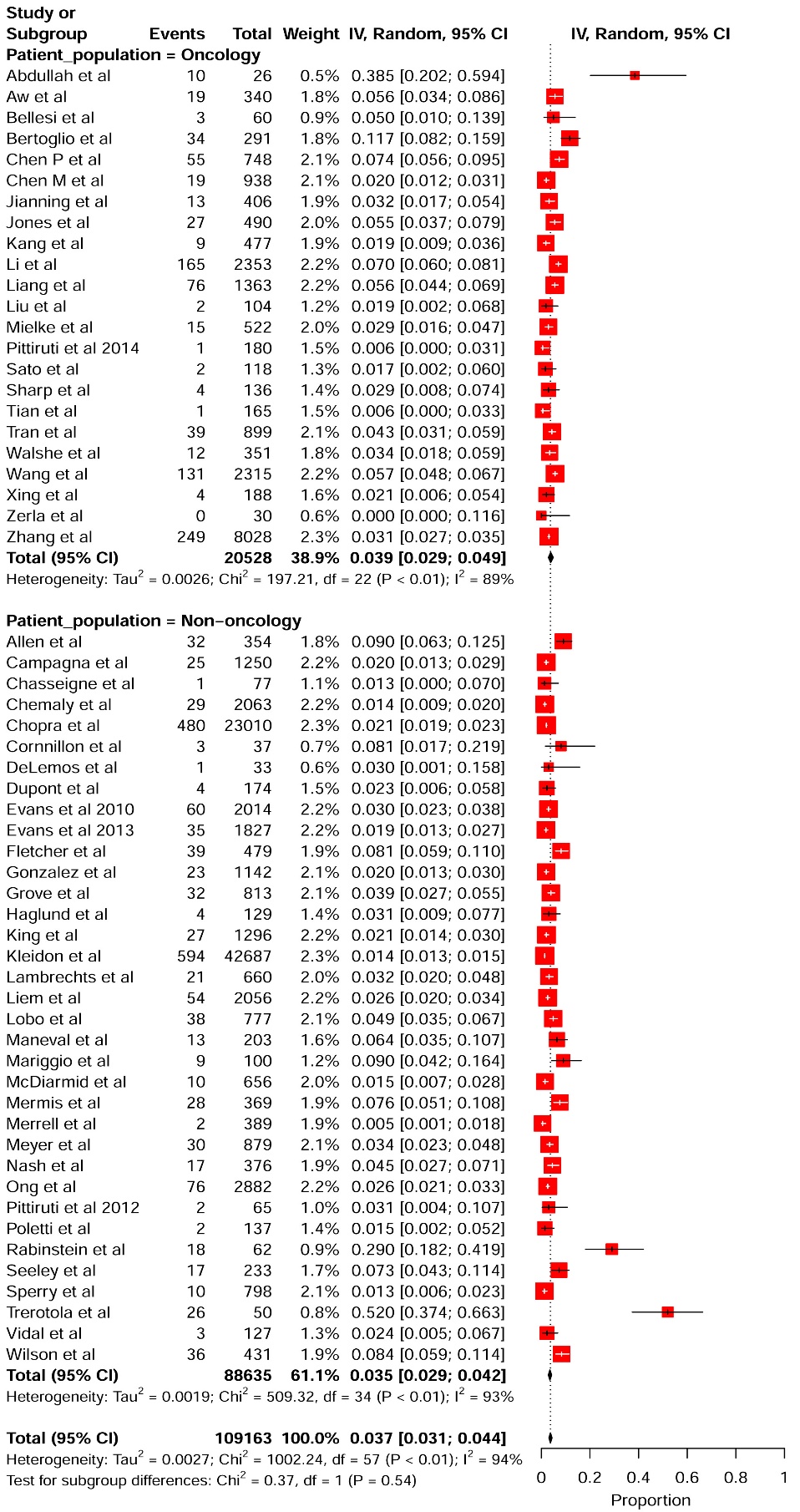
**

**Supplementary Figure 5. Forest plot showing the incidence of VTE associated with PICC based on the type of VTE**

**
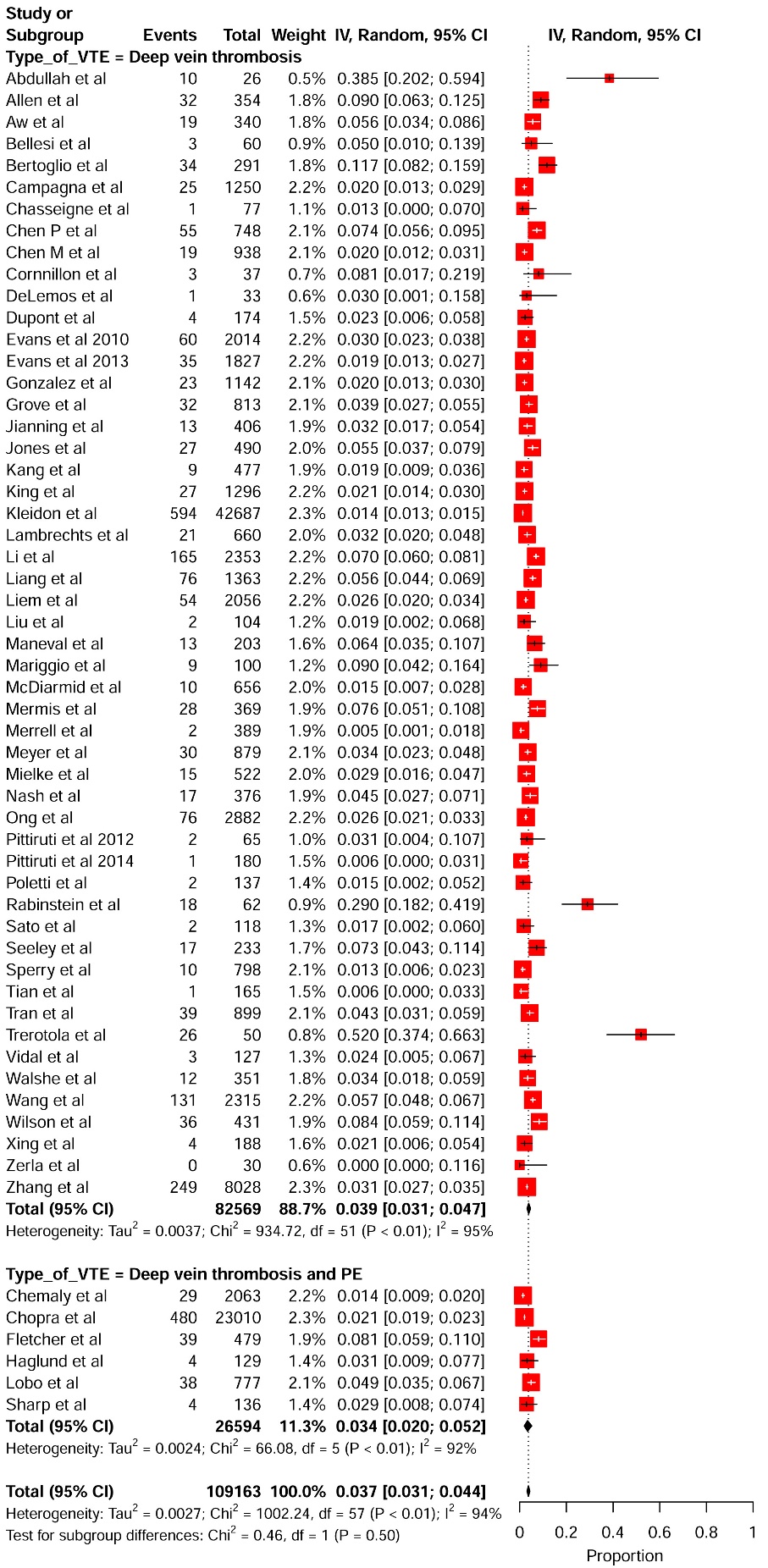
**

**Supplementary Figure 6. Forest plot for the incidence of VTE associated with PICC stratified by DVT prophylaxis**

**
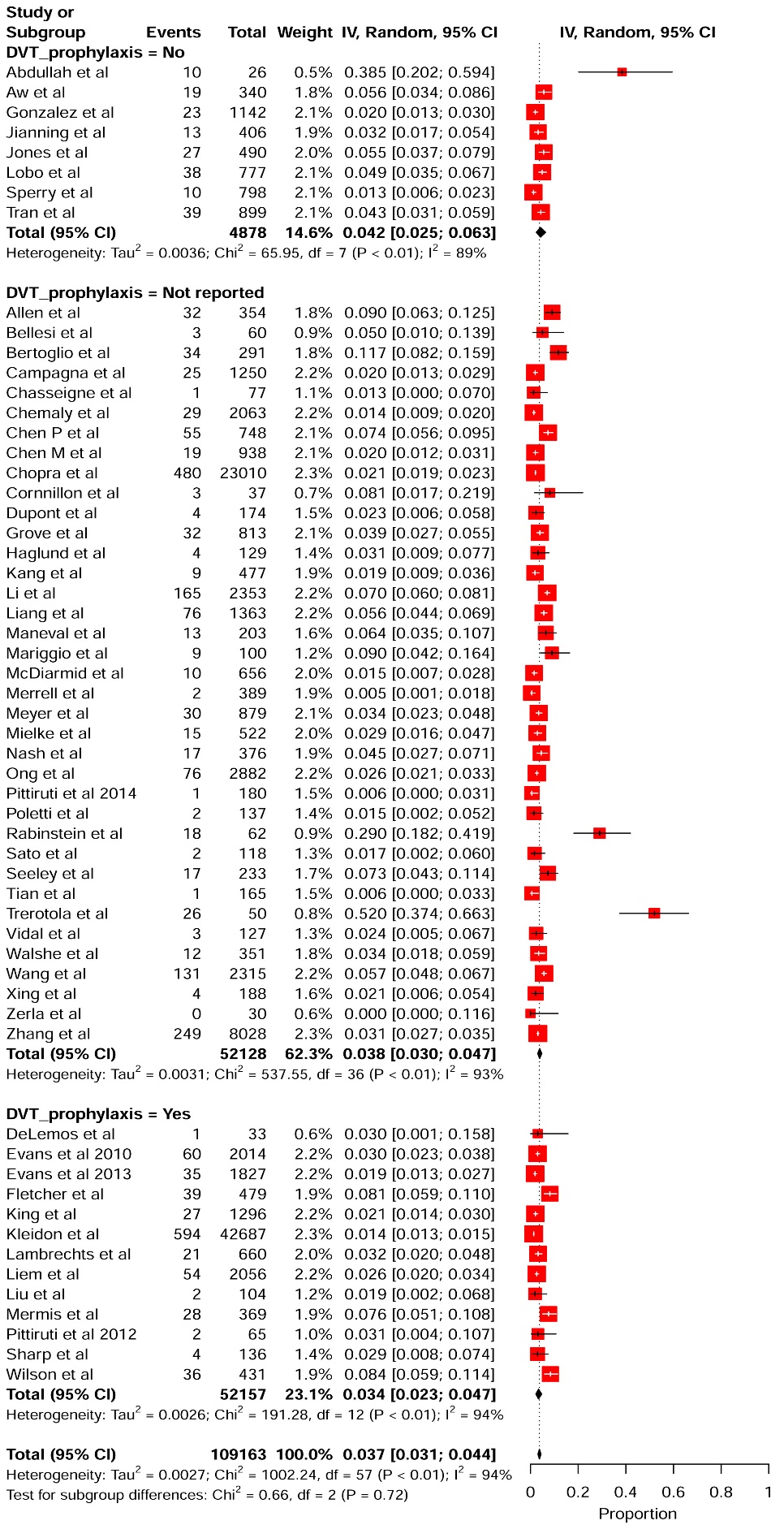
**

**Supplementary Figure 7. Forest plot depicting the incidence of PICC-related VTE stratified by study design**

**
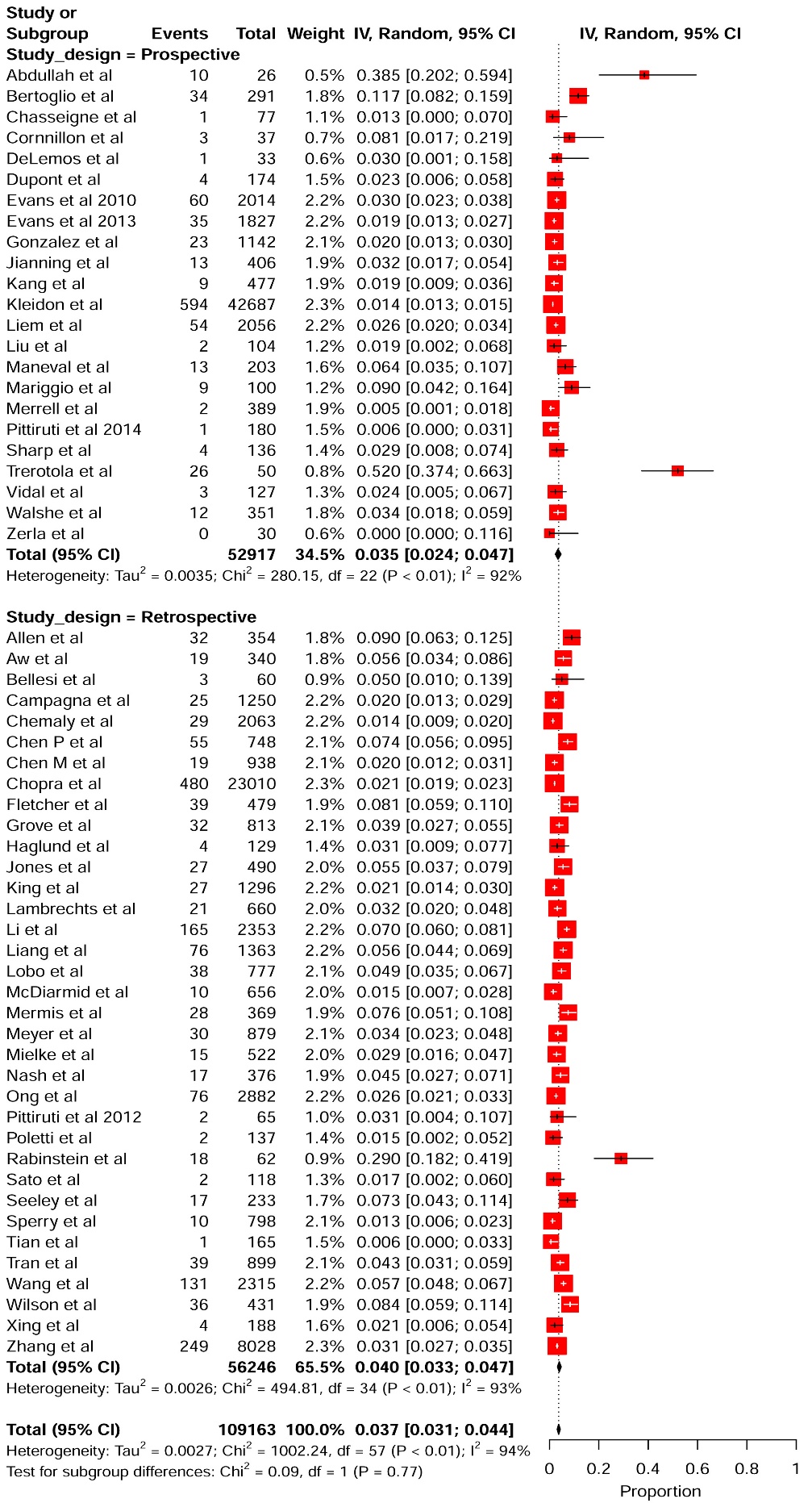
**

**Supplementary Figure 8**. **Forest plot depicting the incidence of PICC-related VTE according to study location**

**
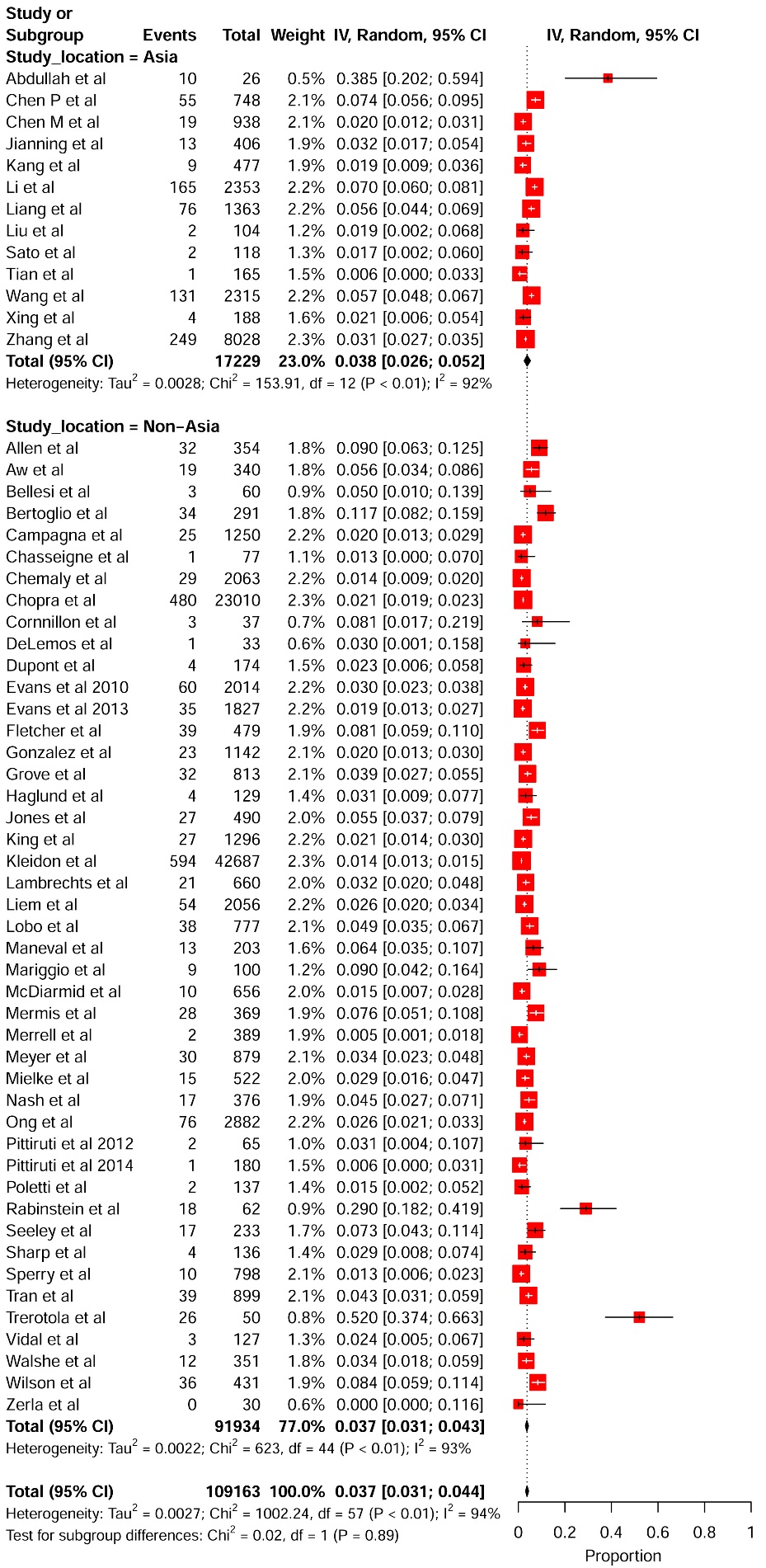
**

**Supplementary Figure 9.** **Forest plot showing the incidence of VTE associated with PICC, stratified by publication year**

**
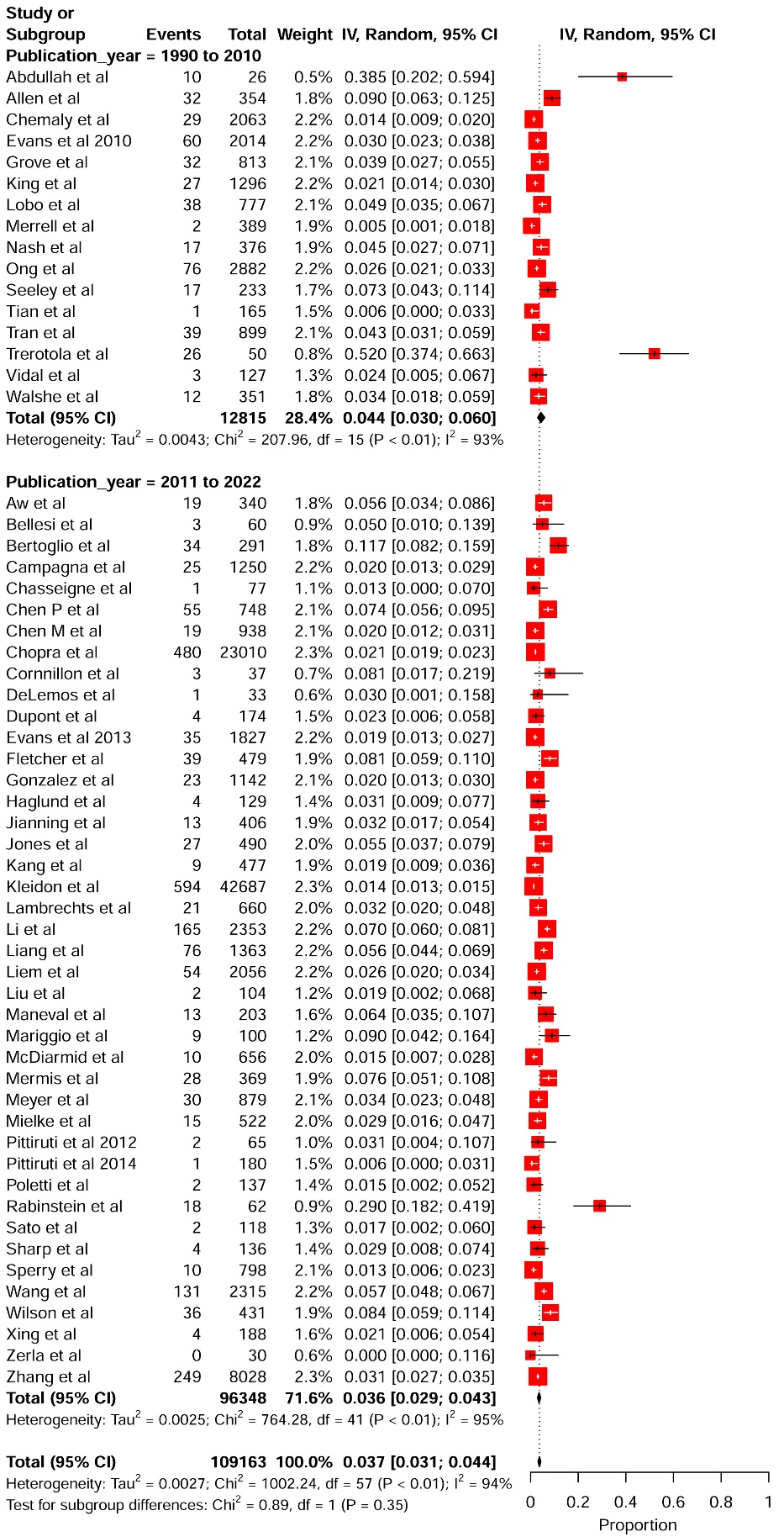
**

**Supplementary Figure 10. Funnel plot to detect publication bias for studies comparing VTE risk between PICC and CVC**. A nearly symmetrical funnel plot was obtained, indicating that there was no evidence of publication bias.

**
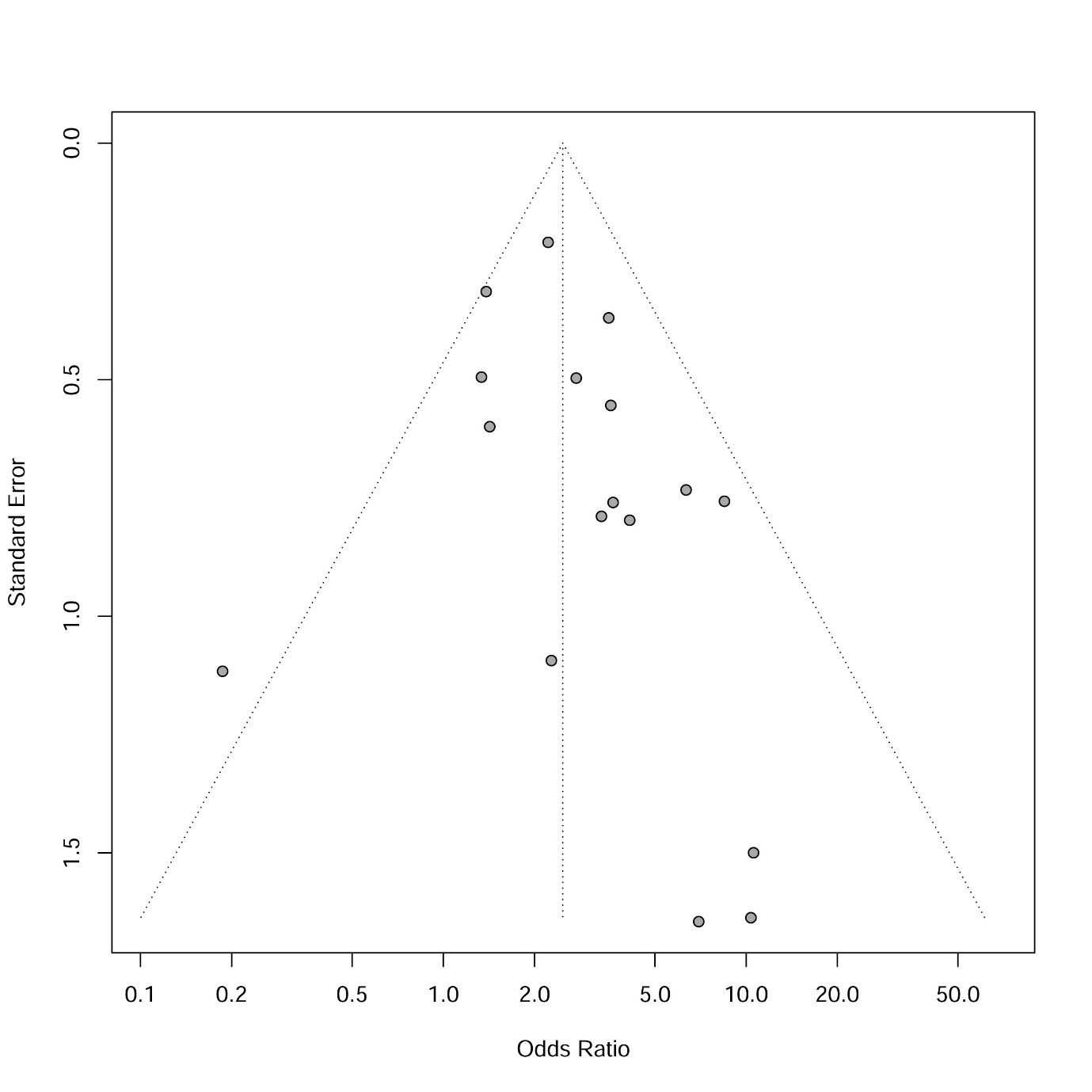
**

**Supplementary Figure 11**. **Forest plot showing the risk of VTE associated with PICC based on the study setting (Non-critical care/non-ICU vs. Critical care/ICU)**


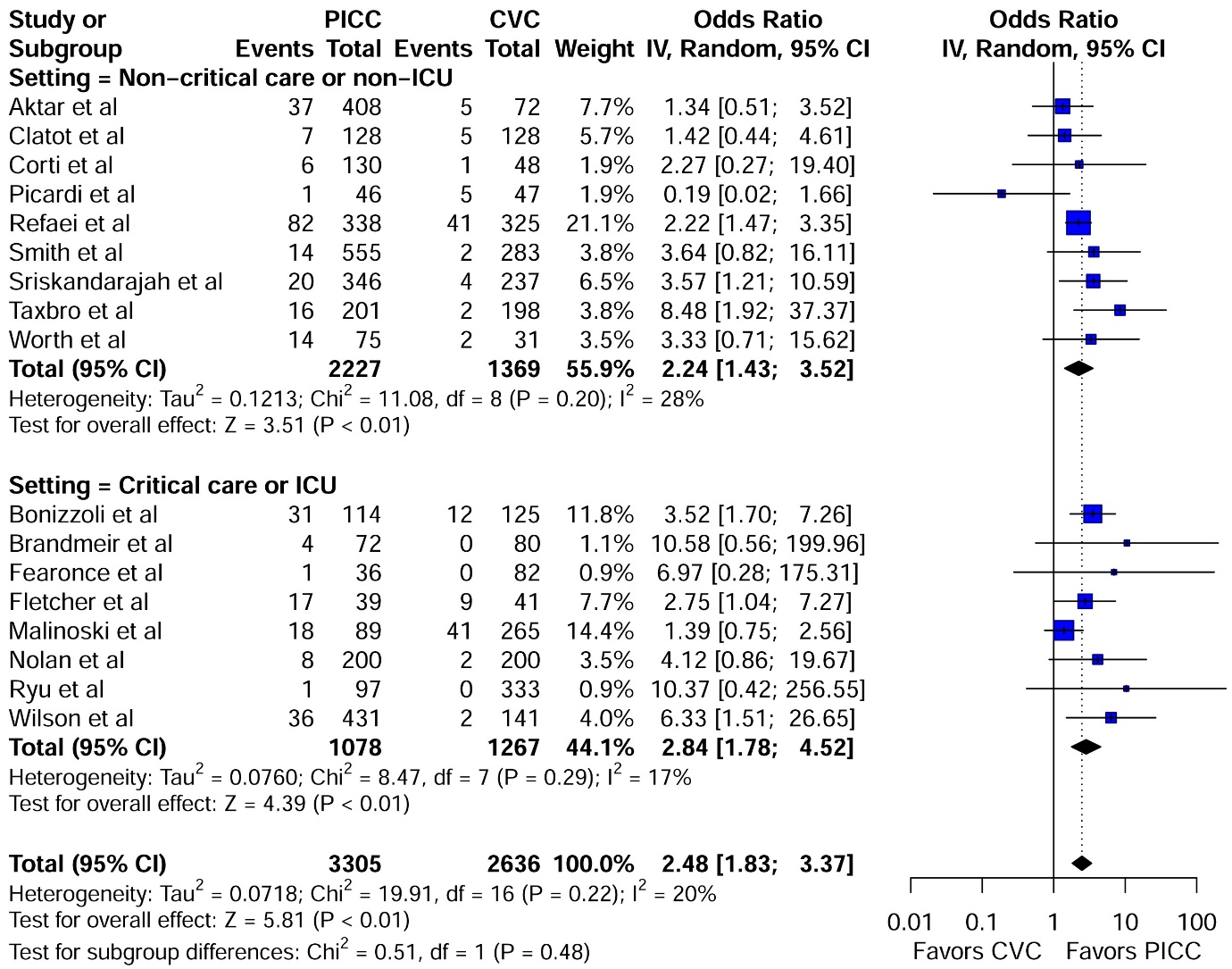


**Supplementary Figure 12. Forest plot showing the risk of VTE associated with PICC stratified by patient population (Oncology vs. non-oncology patients)**


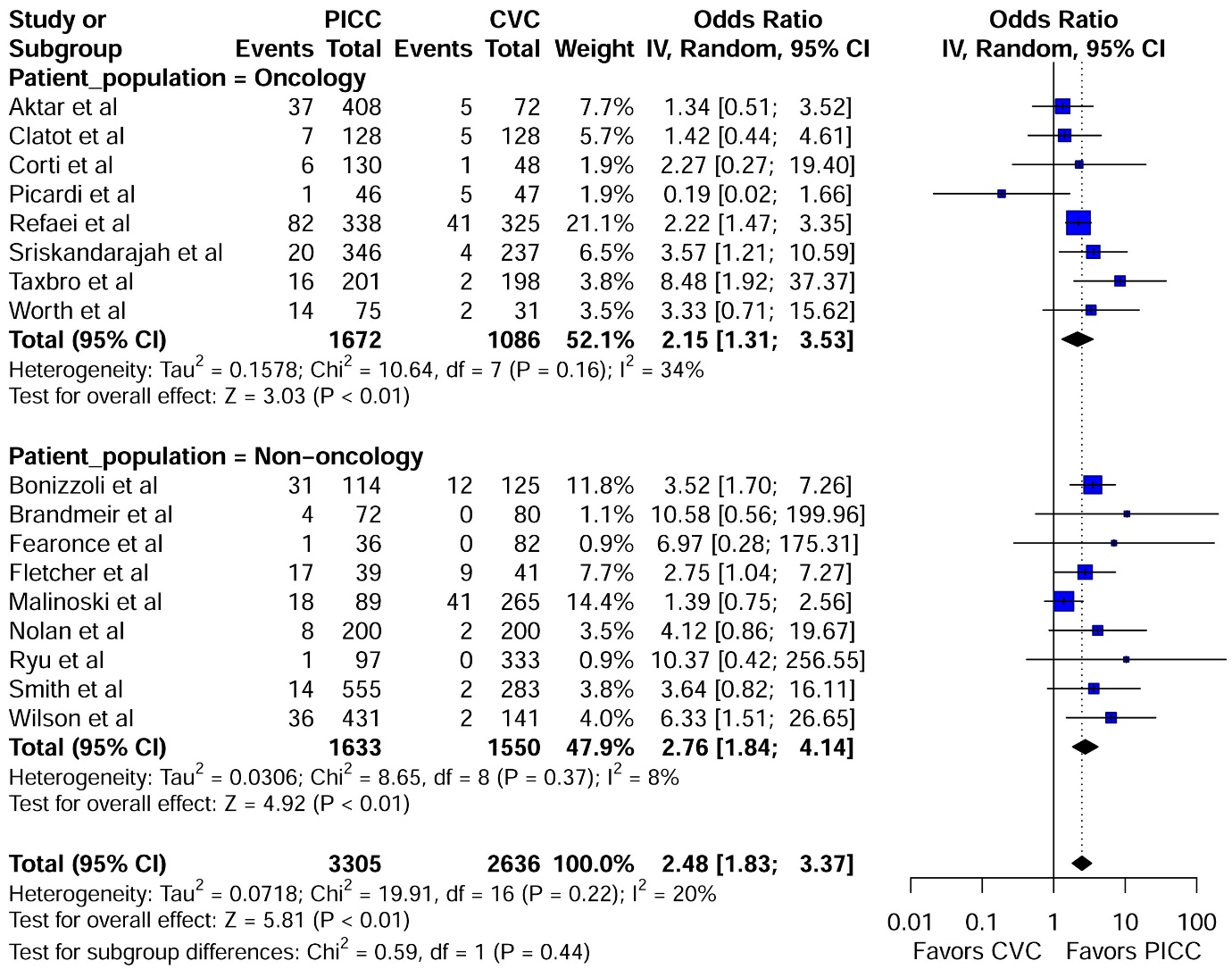


**Supplementary Figure 13. Forest plot showing the risk of VTE associated with PICC stratified by DVT prophylaxis (DVT prophylaxis not reported vs. DVT prophylaxis reported)**


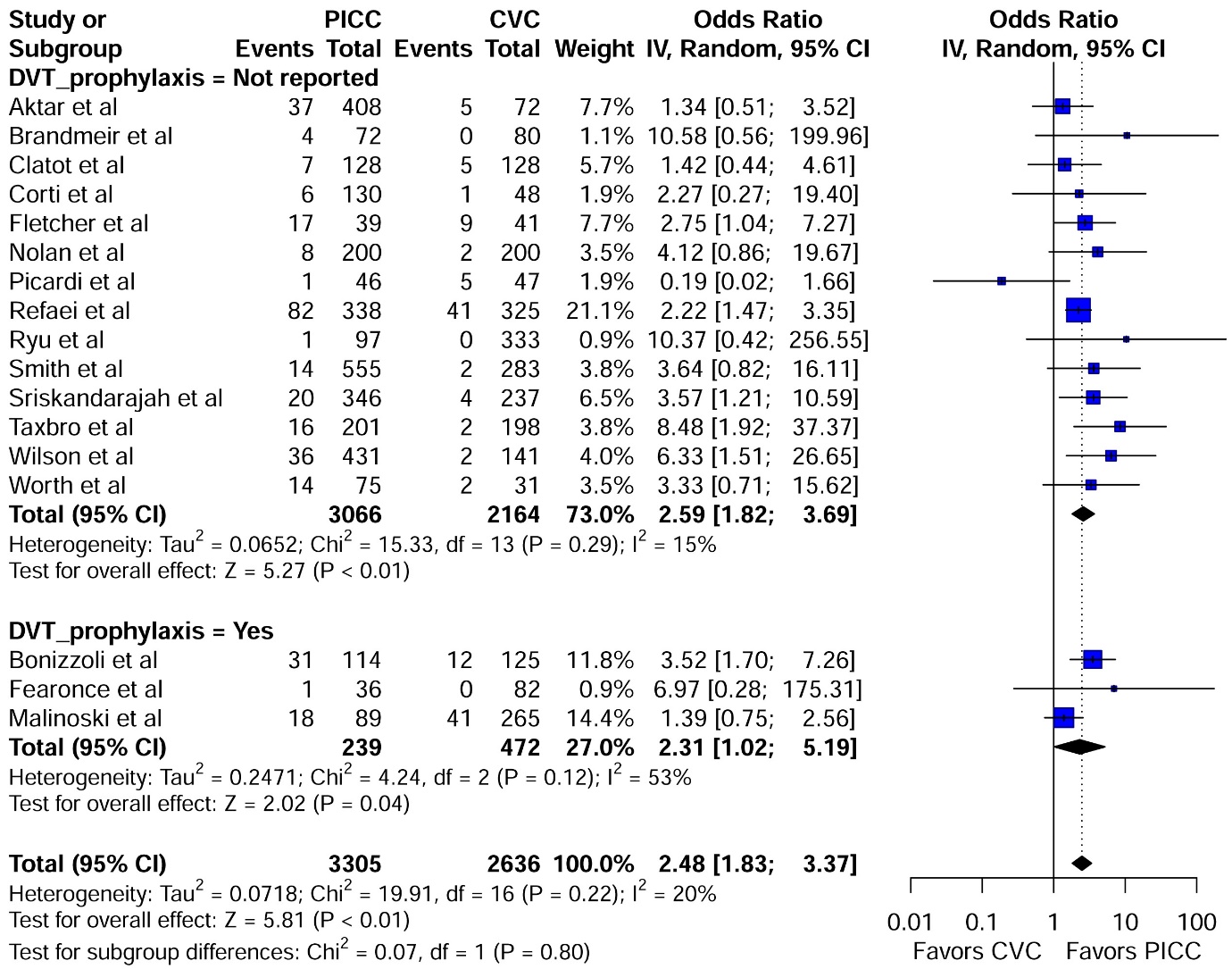


**Supplementary Figure 14. Forest plot depicting the risk of PICC-related VTE associated stratified by study design (Retrospective vs. prospective vs. randomized controlled trials)**


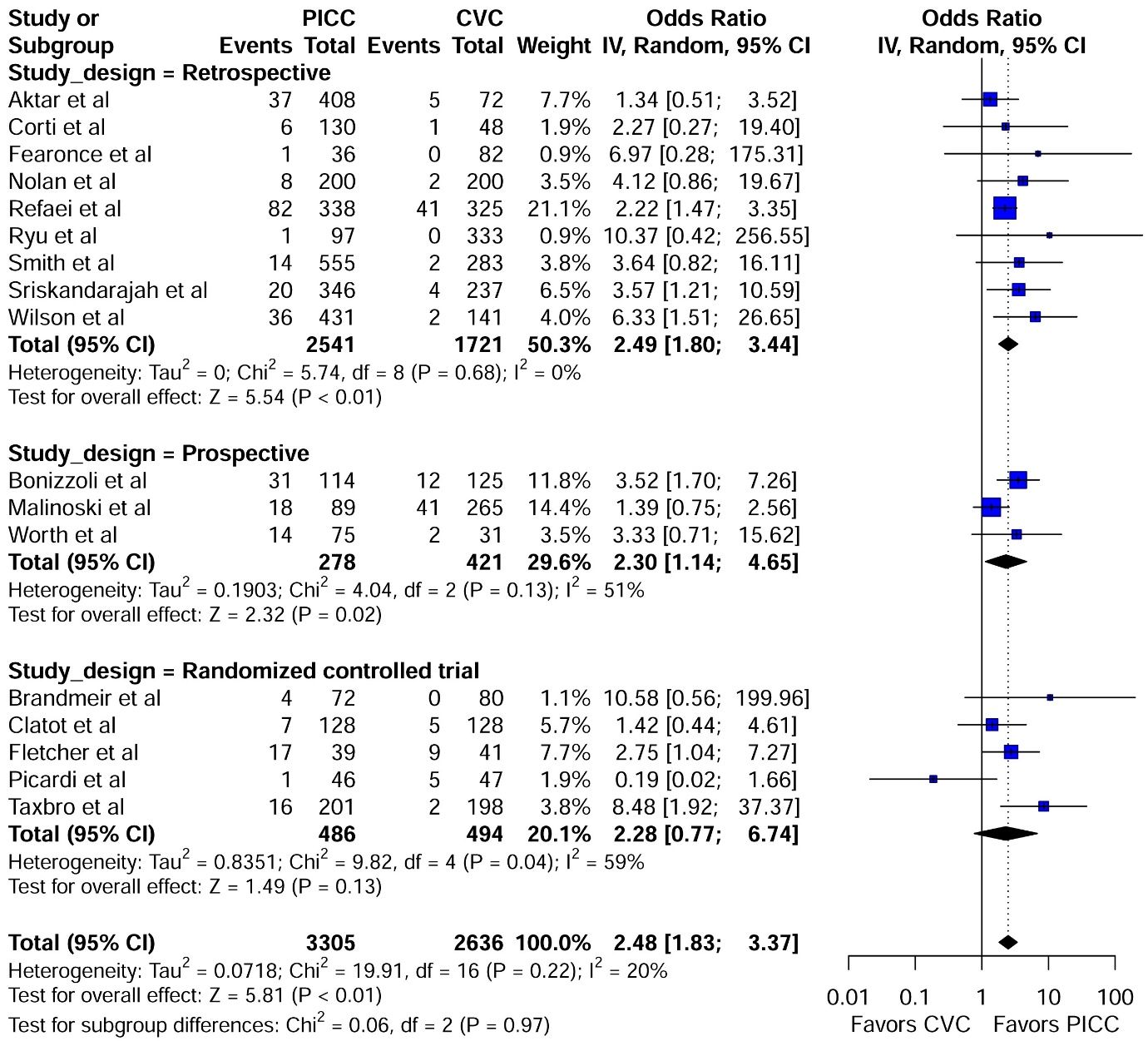


**Supplementary Figure 15. Forest plot depicting the risk of PICC-related VTE associated stratified by study location (studies conducted outside America vs. studies conducted in America)**


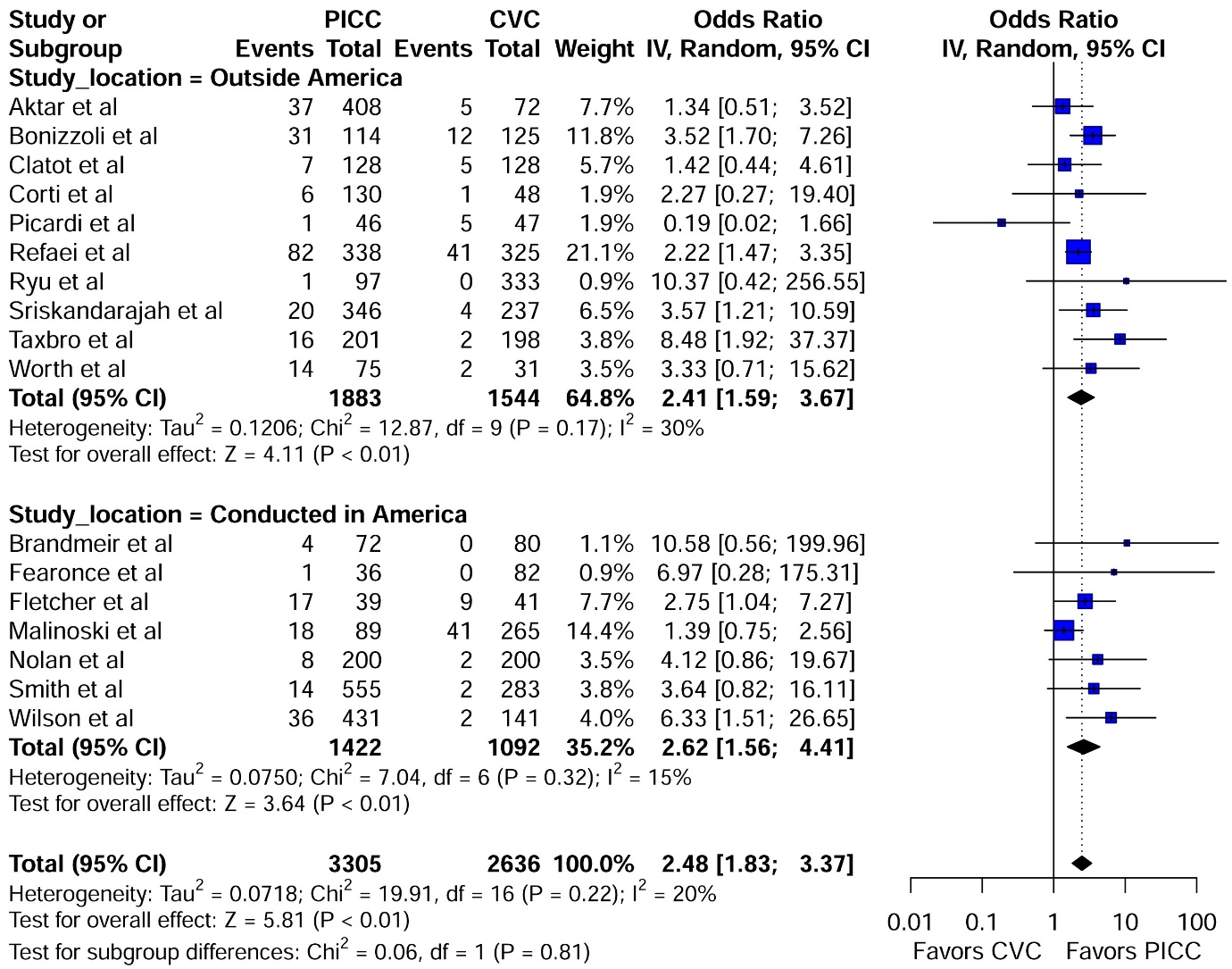


**Supplementary Figure 16. Forest plot depicting the risk of PICC-related VTE associated stratified by publication year (studies published from 2011 to 2022 vs. studies published from 1990 to 2010)**


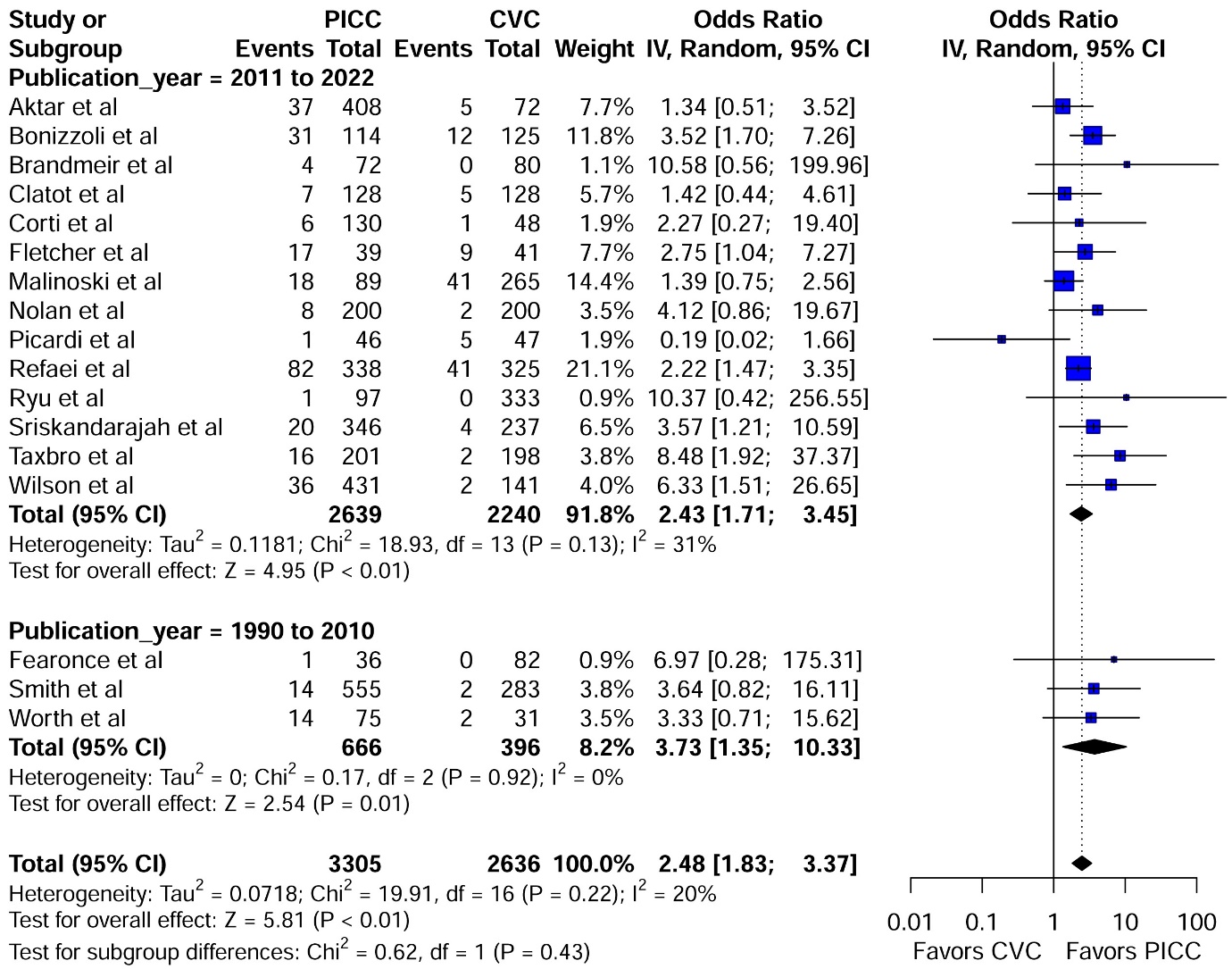


**Supplementary Table 1.** Quality assessments of included studies using the Newcastle-Ottawa scale.

| **Author, year** | **Selection of Exposed and Non-Exposed Cohorts** | | | | **Comparability** | **Outcome of Interest** | | | **Overall**  **Quality** |
| --- | --- | --- | --- | --- | --- | --- | --- | --- | --- |
|  | Representativeness  of exposed cohort | Selection of non-  exposed cohort | Ascertainment of exposure | Outcome present at  start of study | Comparability of cohorts | Assessment of outcome | Length of  follow-up | Adequacy of  follow-up |  |
| **STUDIES WITHOUT A COMPARISON-ARM** | | | | | | | | | |
| Abdullah et al., 2005 | ★ | NA | ★ | ★ | NA | ★ | ★ | ★ | High |
| Allen et al., 2000 | ★ | NA | ★ | ★ | NA | ★ | ★ | ★ | High |
| Aw et al., 2012 | ★ | NA | ★ | ★ | NA | ★ | ★ | ★ | High |
| Bellesi et al., 2013 | ★ | NA | ★ | ★ | NA | ★ | ★ | ★ | High |
| Bertoglio et al., 2016 | ★ | NA | ★ | ★ | NA | ★ | ★ | ★ | High |
| Campagna et al., 2019 | ★ | NA | ★ | ★ | NA | ★ | ★ | ★ | High |
| Chasseigne et al., 2020 | ★ | NA | ★ | ★ | NA | ★ | ★ | ★ | High |
| Chemaly et al., 2002 | ★ | NA | ★ | ★ | NA | ★ | ★ | ★ | High |
| Chen et al. 2021 | ★ | NA | ★ | ★ | NA | ★ | ★ | ★ | High |
| Chen et al. 2020 | ★ | NA | ★ | ★ | NA | ★ | ★ | ★ | High |
| Chopra et al., 2018 | ★ | NA | ★ | ★ | NA | ★ | ★ | ★ | High |
| Cornnillon et al., 2017 | ★ | NA | ★ | ★ | NA | ★ | ★ | ★ | High |
| DeLemos et al., 2011 | ★ | NA | NR | ★ | NA | NR | ★ | ★ | Moderate |
| Dupont et al., 2015 | ★ | NA | ★ | ★ | NA | ★ | ★ | ★ | High |
| Evans et al., 2010 | ★ | NA | ★ | ★ | NA | ★ | ★ | ★ | High |
| Evans et al., 2013 | ★ | NA | ★ | ★ | NA | ★ | ★ | ★ | High |
| Fletcher et al., 2011 | ★ | NA | ★ | ★ | NA | ★ | ★ | ★ | High |
| Gonzalez et al. 2021 | ★ | NA | ★ | ★ | NA | ★ | ★ | ★ | High |
| Grove et al., 2000 | ★ | NA | ★ | ★ | NA | ★ | ★ | ★ | High |
| Haglund et al., 2014 | ★ | NA | ★ | ★ | NA | ★ | ★ | ★ | High |
| Jianning et al., 2018 | ★ | NA | ★ | ★ | NA | ★ | ★ | ★ | High |
| Jones et al., 2017 | ★ | NA | ★ | ★ | NA | ★ | ★ | ★ | High |
| Kang et al., 2017 | ★ | NA | ★ | ★ | NA | NR | ★ | ★ | Moderate |
| King et al., 2006 | ★ | NA | ★ | ★ | NA | ★ | NR | ★ | Moderate |
| Kleidon et al. 2021 | ★ | NA | ★ | ★ | NA | ★ | ★ | ★ | High |
| Lambrechts et al. 2021 | ★ | NA | ★ | ★ | NA | ★ | ★ | ★ | High |
| Li et al., 2021 | ★ | NA | ★ | ★ | NA | ★ | ★ | ★ | High |
| Liang et al., 2018 | ★ | NA | ★ | ★ | NA | ★ | ★ | ★ | High |
| Liem et al., 2012 | ★ | NA | ★ | ★ | NA | ★ | ★ | ★ | High |
| Liu et al., 2018 | ★ | NA | ★ | ★ | NA | ★ | ★ | ★ | High |
| Lobo et al., 2009 | ★ | NA | ★ | ★ | NA | ★ | ★ | ★ | High |
| Maneval et al., 2014 | ★ | NA | ★ | ★ | NA | ★ | ★ | ★ | High |
| Mariggio et al., 2020 | ★ | NA | ★ | ★ | NA | ★ | ★ | ★ | High |
| McDiarmid et al., 2017 | ★ | NA | ★ | ★ | NA | NR | ★ | ★ | Moderate |
| Mermis et al., 2014 | ★ | NA | ★ | ★ | NA | ★ | ★ | ★ | High |
| Merrell et al., 1994 | ★ | NA | ★ | ★ | NA | ★ | ★ | ★ | High |
| Meyer et al., 2011 | ★ | NA | ★ | ★ | NA | ★ | ★ | ★ | High |
| Mielke et al., 2020 | ★ | NA | ★ | ★ | NA | ★ | ★ | ★ | High |
| Nash et al., 2009 | ★ | NA | ★ | ★ | NA | ★ | ★ | ★ | High |
| Ong et al., 2006 | ★ | NA | ★ | ★ | NA | ★ | NR | ★ | Moderate |
| Pittiruti et al., 2012 | ★ | NA | ★ | ★ | NA | ★ | ★ | ★ | High |
| Pittiruti et al., 2014 | ★ | NA | ★ | ★ | NA | ★ | ★ | ★ | High |
| Poletti et al., 2014 | ★ | NA | ★ | ★ | NA | ★ | ★ | ★ | High |
| Rabinstein et al., 2020 | ★ | NA | ★ | ★ | NA | ★ | ★ | NR | Moderate |
| Sato et al., 2021 | ★ | NA | ★ | ★ | NA | ★ | ★ | ★ | High |
| Seeley et al., 2007 | ★ | NA | NR | ★ | NA | ★ | ★ | ★ | Moderate |
| Sharp et al., 2015 | ★ | NA | ★ | ★ | NA | ★ | ★ | ★ | High |
| Sperry et al., 2012 | ★ | NA | ★ | ★ | NA | ★ | ★ | ★ | High |
| Tian et al., 2010 | ★ | NA | ★ | ★ | NA | NR | ★ | ★ | Moderate |
| Tran et al., 2010 | ★ | NA | ★ | ★ | NA | ★ | ★ | ★ | High |
| Trerotola et al., 2010 | ★ | NA | ★ | ★ | NA | ★ | ★ | ★ | High |
| Vidal et al., 2008 | ★ | NA | NR | NR | NA | ★ | ★ | ★ | Moderate |
| Walshe et al., 2002 | ★ | NA | ★ | ★ | NA | ★ | ★ | ★ | High |
| Wang et al., 2021 | ★ | NA | ★ | ★ | NA | ★ | ★ | ★ | High |
| Wilson et al., 2012 | ★ | NA | ★ | ★ | NA | ★ | ★ | ★ | High |
| Xing et al., 2011 | ★ | NA | ★ | ★ | NA | ★ | ★ | ★ | High |
| Zerla et al., 2017 | ★ | NA | ★ | ★ | NA | ★ | ★ | ★ | High |
| Zhang et al., 2016 | ★ | NA | ★ | ★ | NA | ★ | ★ | ★ | High |
| **STUDIES WITH A COMPARISON-ARM** | | | | | | | | | |
| Aktar et al., 2021 | ★ | ★ | ★ | ★ | ★ | ★ | ★ | ★ | High |
| Bonizzoli et al., 2011 | ★ | ★ | ★ | ★ | ★ | ★ | ★ | ★ | High |
| Brandmeir et al., 2019 | ★ | ★ | ★ | ★ | ★ | ★ | ★ | ★ | High |
| Clatot et al., 2019 | ★ | ★ | ★ | ★ | ★ | ★ | ★ | ★ | High |
| Corti et al., 2021 | ★ | ★ | ★ | ★ | ★ | ★ | ★ | ★ | High |
| Fearonce et al., 2010 | ★ | ★ | ★ | ★ | ★ | ★ | ★ | ★ | High |
| Fletcher et al., 2016 | ★ | ★ | ★ | ★ | ★ | ★ | ★ | ★ | High |
| Malinoski et al., 2013 | ★ | ★ | ★ | ★ | ★ | ★ | ★ | ★ | High |
| Nolan et al., 2016 | ★ | ★ | ★ | ★ | ★ | ★ | ★ | ★ | High |
| Picardi et al., 2018 | ★ | ★ | ★ | ★ | ★ | ★ | ★ | ★ | High |
| Refaei et al., 2016 | ★ | ★ | ★ | ★ | ★ | ★ | ★ | ★ | High |
| Ryu et al., 2019 | ★ | ★ | ★ | ★ | ★ | ★ | ★ | ★ | High |
| Smith et al., 1998 | ★ | ★ | ★ | ★ | ★ | ★ | ★ | ★ | High |
| Sriskandarajah et al., 2015 | ★ | ★ | ★ | ★ | ★ | ★ | ★ | ★ | High |
| Taxbro et al., 2019 | ★ | ★ | ★ | ★ | ★ | ★ | ★ | ★ | High |
| Wilson et al., 2013 | ★ | ★ | ★ | ★ | ★ | ★ | ★ | ★ | High |
| Worth et al., 2009 | ★ | ★ | ★ | ★ | ★ | NR | ★ | ★ | Moderate |

NR, not reported; NA, not applicable
